# Supplementary figures and images for: Coordination of cytochrome bc1 complex assembly at MICOS
Source: EMBO Rep. 2024 Dec 2;26(2):353–84. doi: 10.1038/s44319-024-00336-x (PMC11772845; doi:10.1038/s44319-024-00336-x)

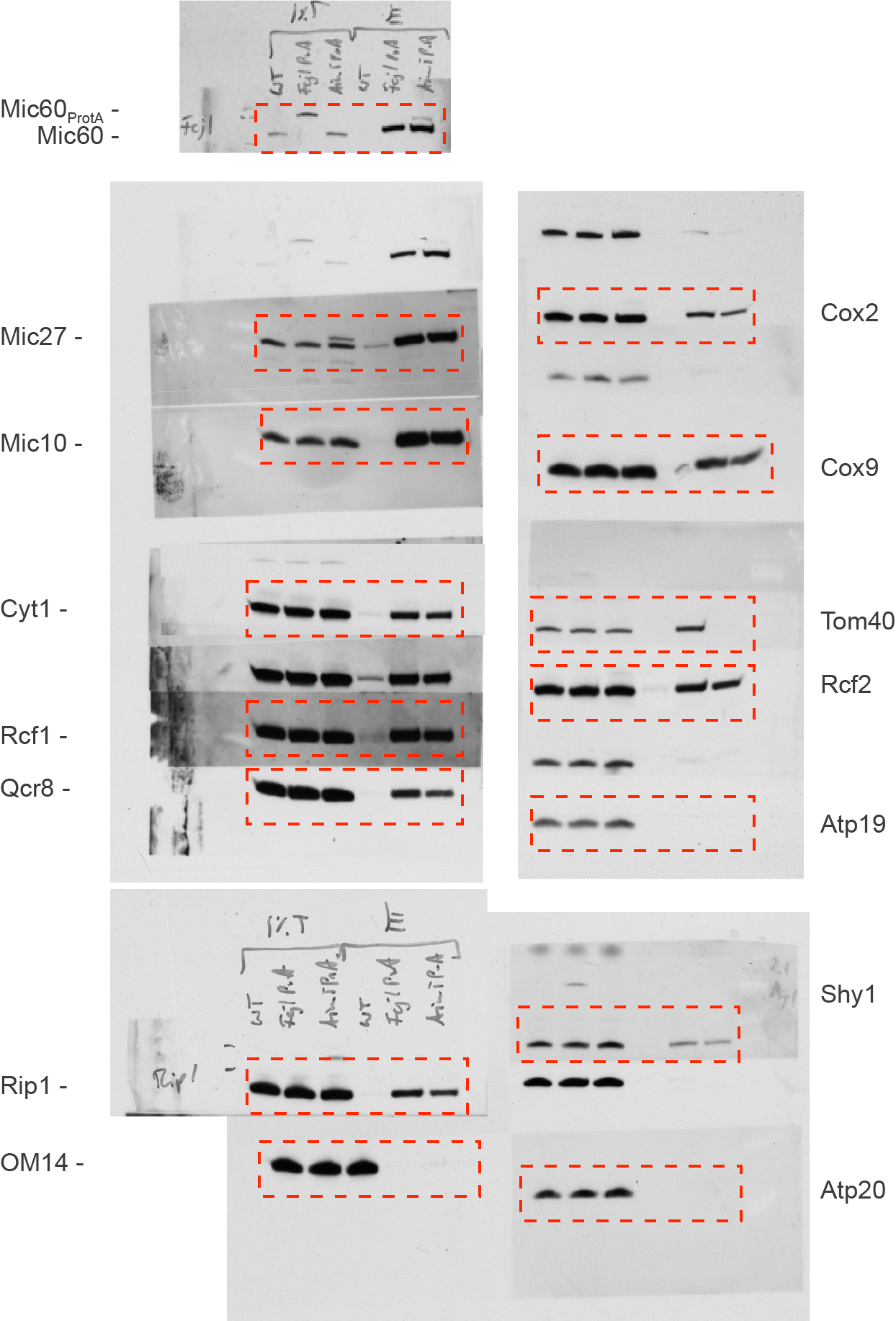

Supplement: Supplementary file 3 — Source data Fig. 1 [file 44319_2024_336_MOESM3_ESM.zip › Zerbes et al - source data Fig 1A.png]

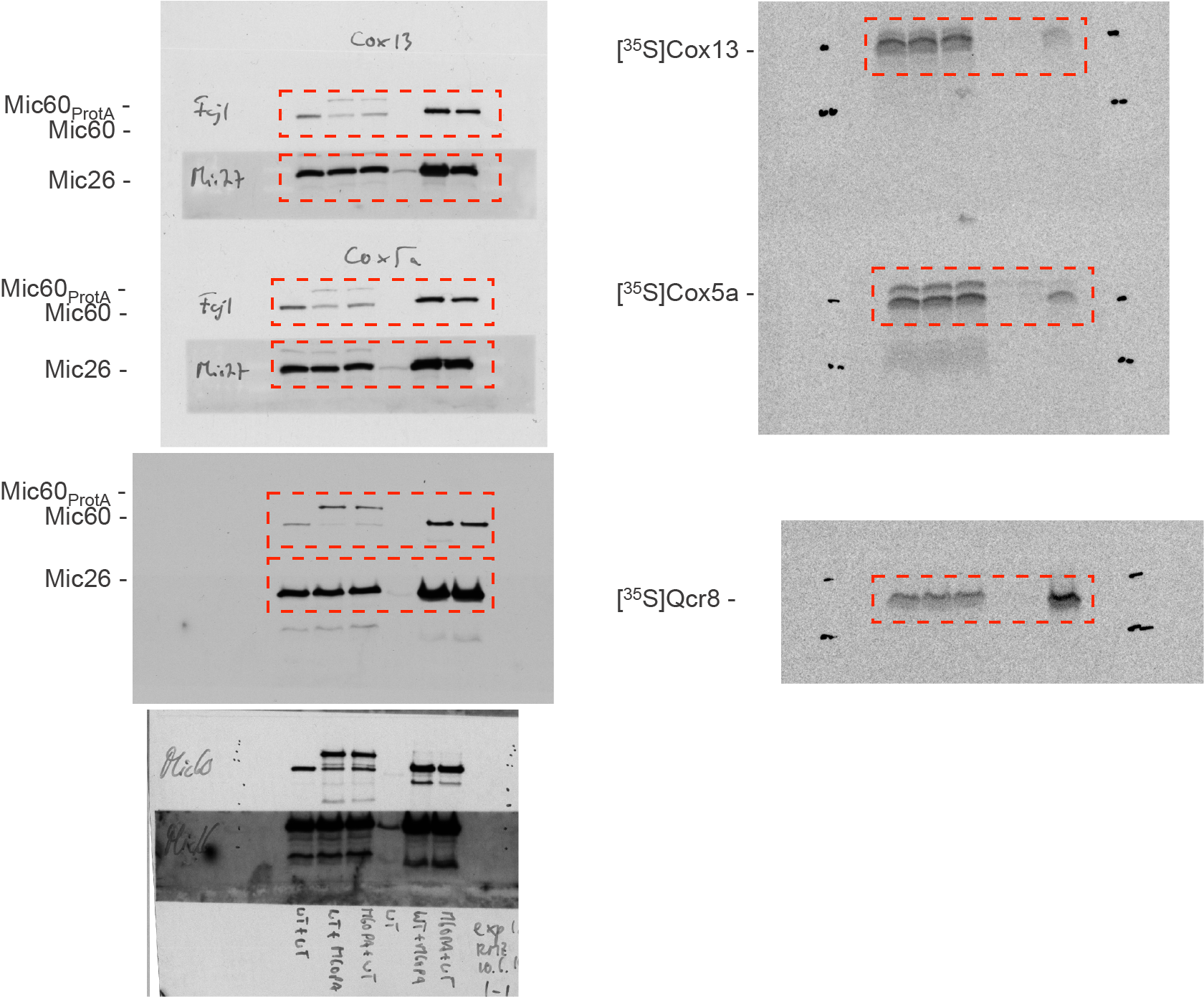

Supplement: Supplementary file 3 — Source data Fig. 1 [file 44319_2024_336_MOESM3_ESM.zip › Zerbes et al - source data Fig 1B.png]

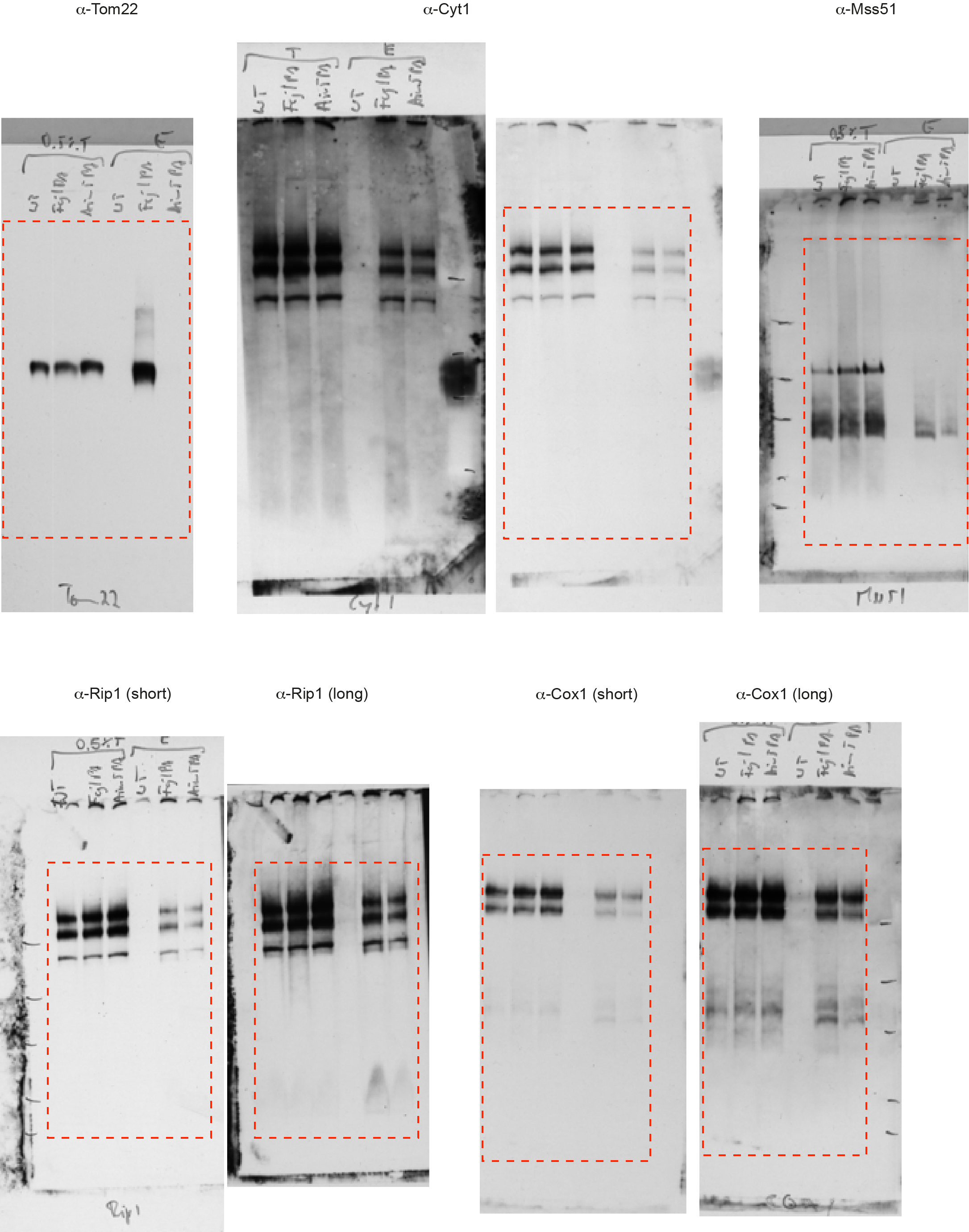

Supplement: Supplementary file 3 — Source data Fig. 1 [file 44319_2024_336_MOESM3_ESM.zip › Zerbes et al - source data Fig 1C.png]

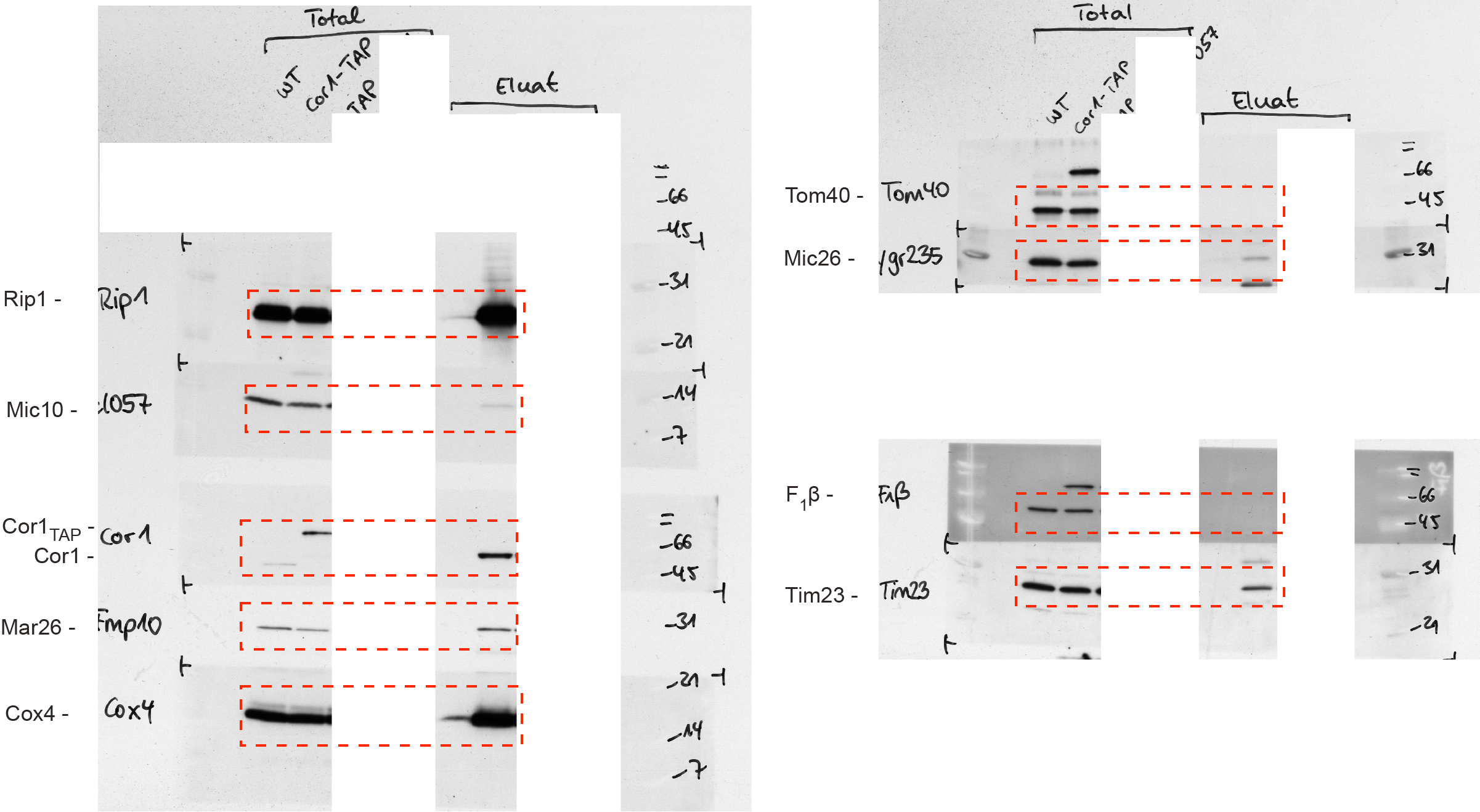

Supplement: Supplementary file 4 — Source data Fig. 2 [file 44319_2024_336_MOESM4_ESM.zip › Zerbes et al - source data Fig 2B.png]

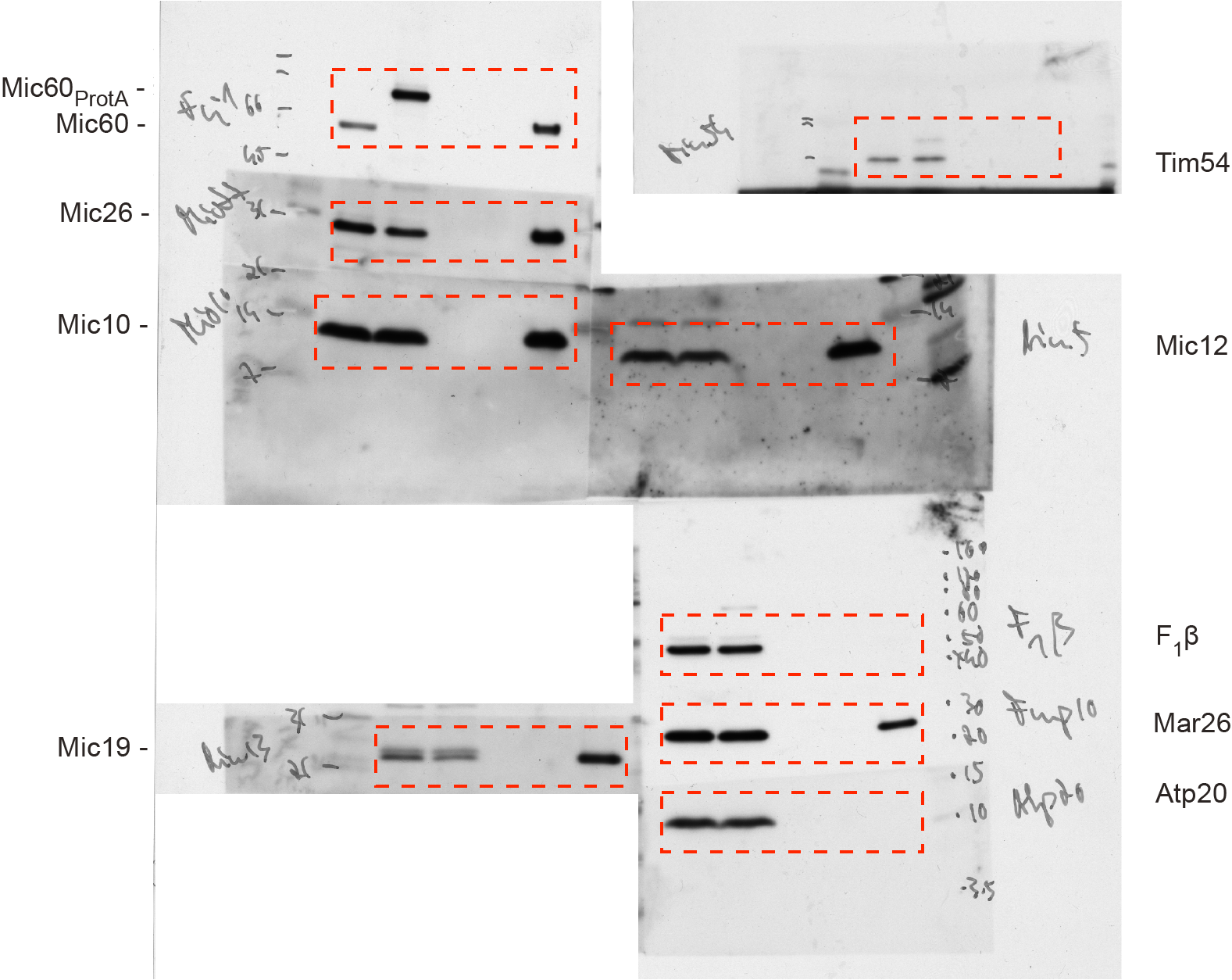

Supplement: Supplementary file 4 — Source data Fig. 2 [file 44319_2024_336_MOESM4_ESM.zip › Zerbes et al - source data Fig 2C.png]

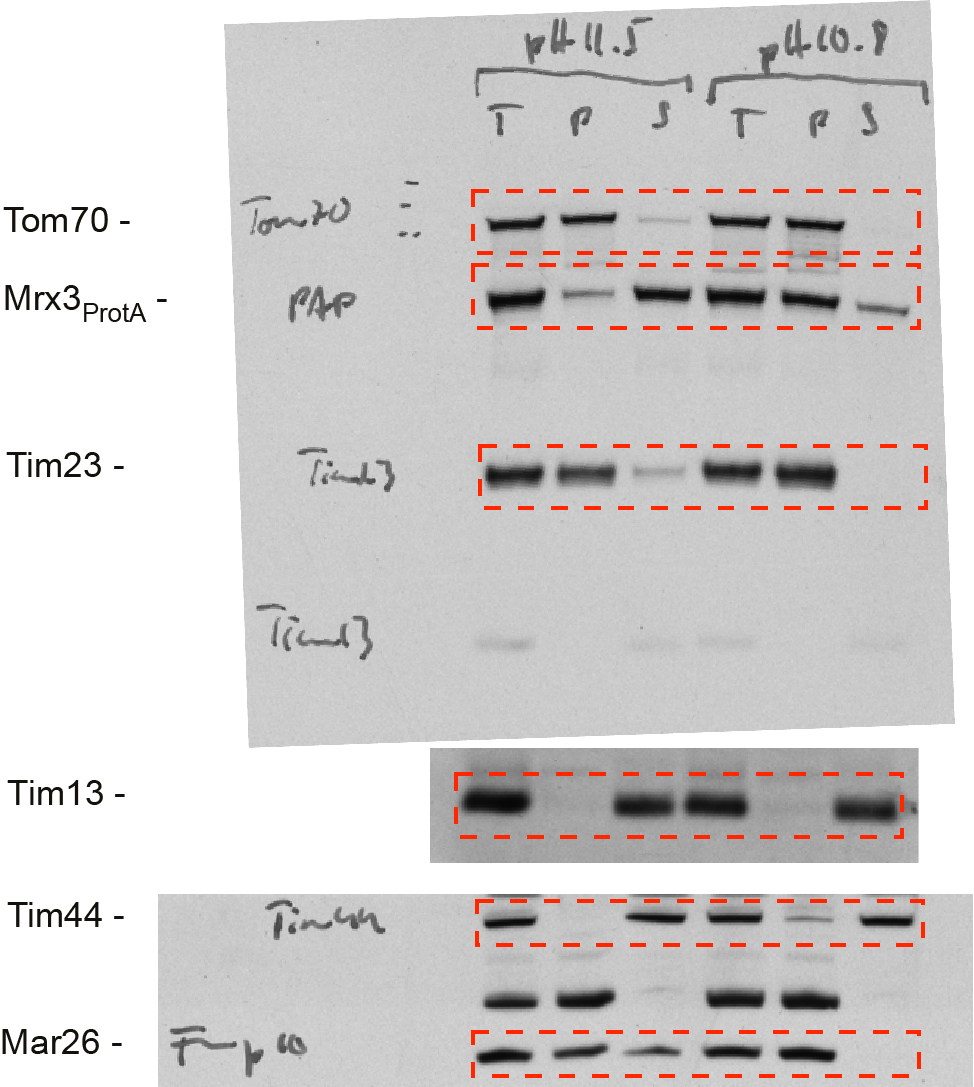

Supplement: Supplementary file 4 — Source data Fig. 2 [file 44319_2024_336_MOESM4_ESM.zip › Zerbes et al - source data Fig 2D.png]

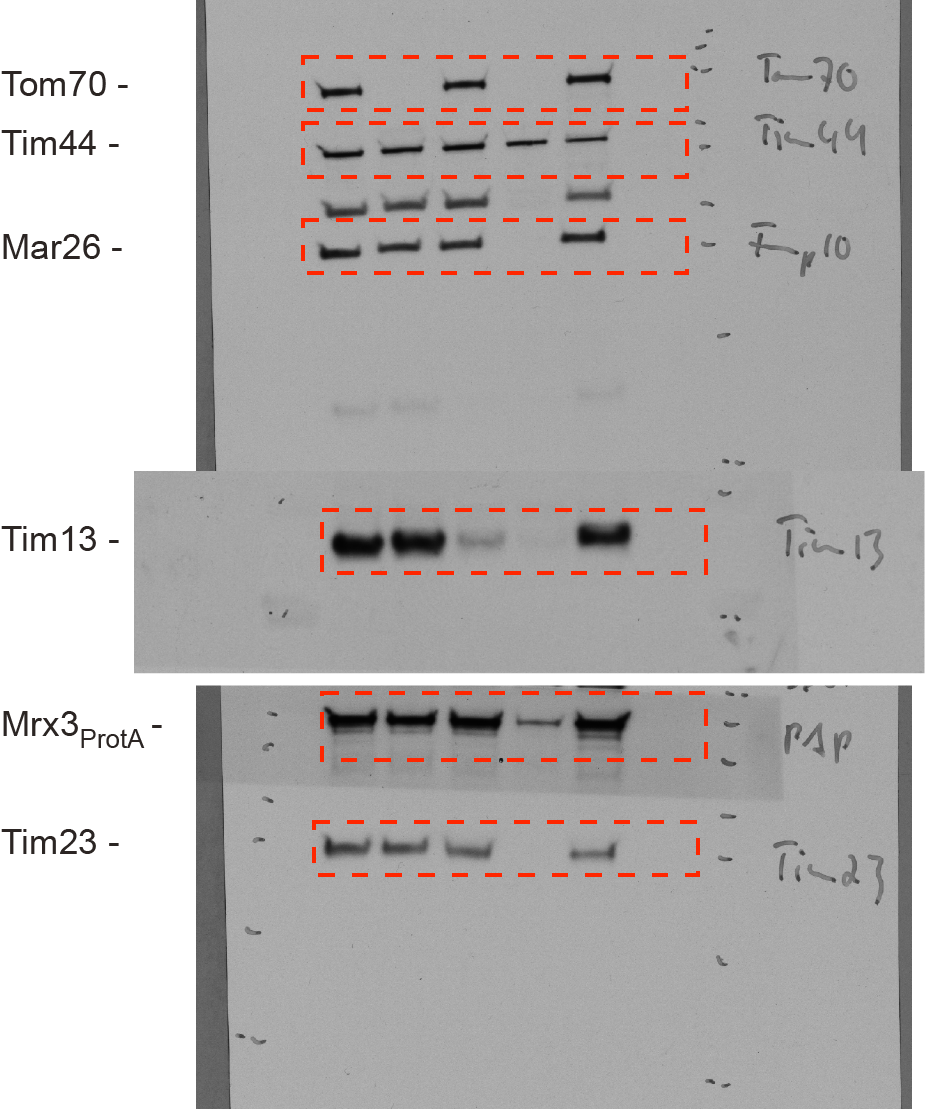

Supplement: Supplementary file 4 — Source data Fig. 2 [file 44319_2024_336_MOESM4_ESM.zip › Zerbes et al - source data Fig 2E.png]

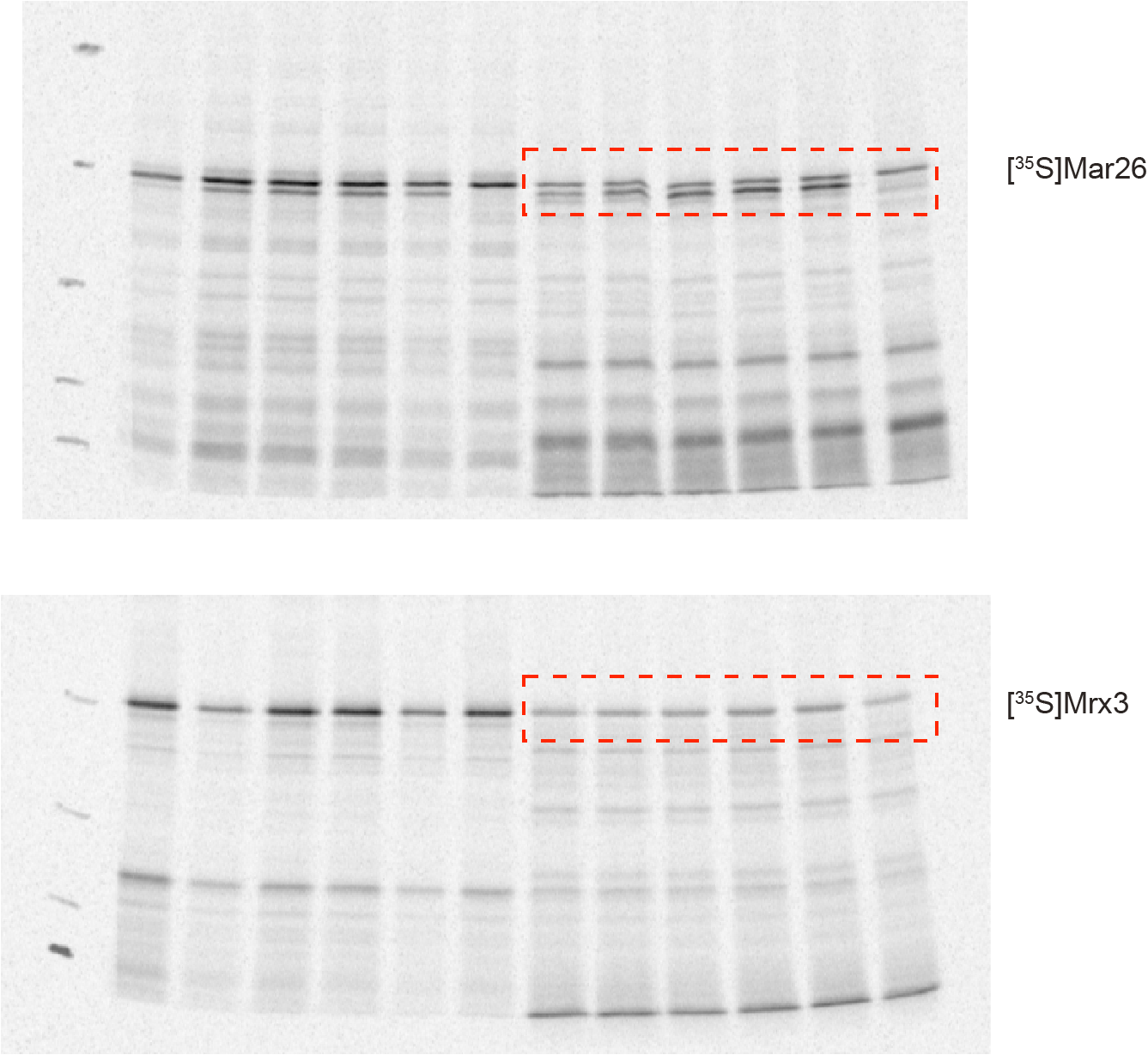

Supplement: Supplementary file 4 — Source data Fig. 2 [file 44319_2024_336_MOESM4_ESM.zip › Zerbes et al - source data Fig 2F.png]

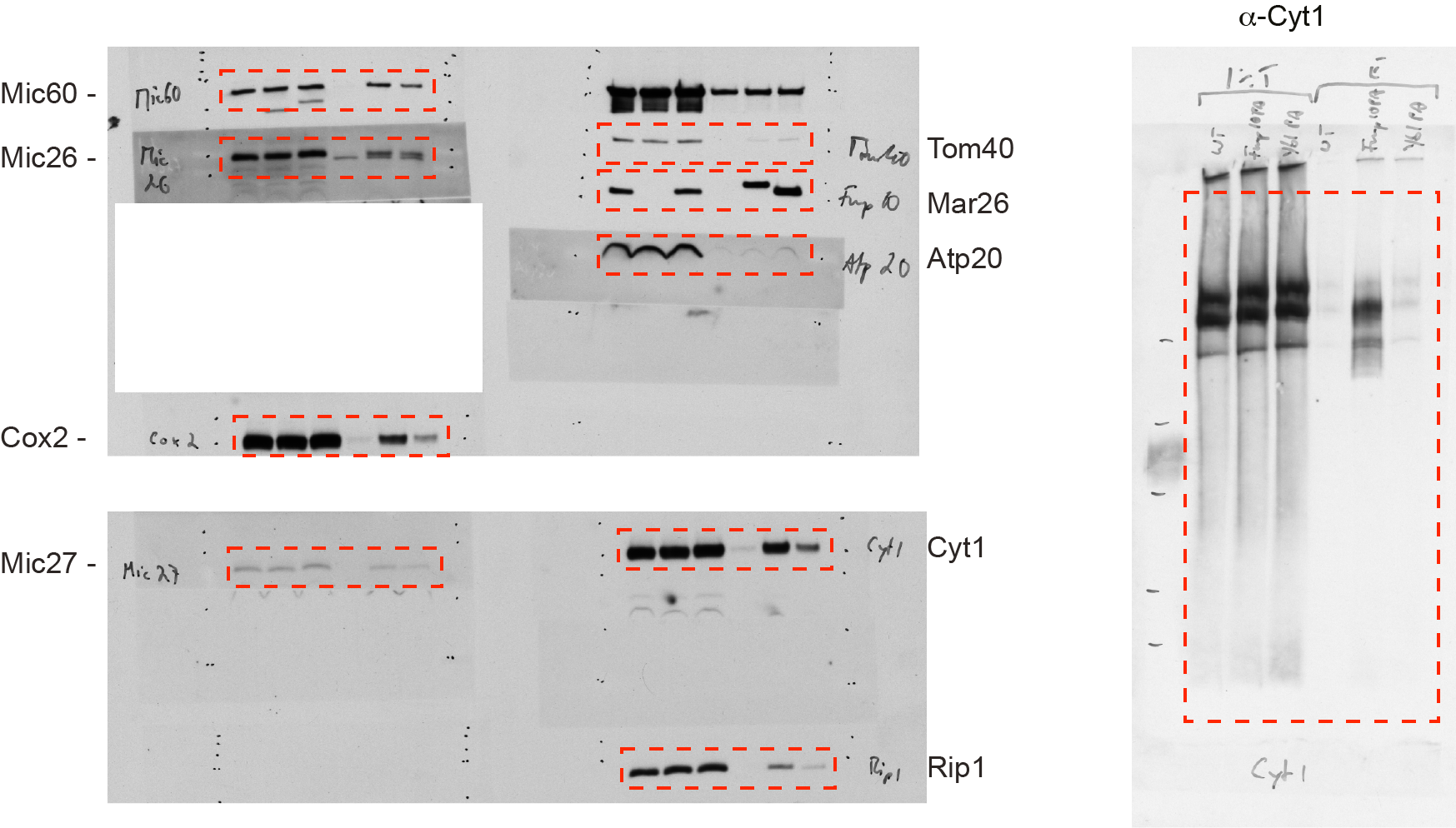

Supplement: Supplementary file 4 — Source data Fig. 2 [file 44319_2024_336_MOESM4_ESM.zip › Zerbes et al - source data Fig 2G.png]

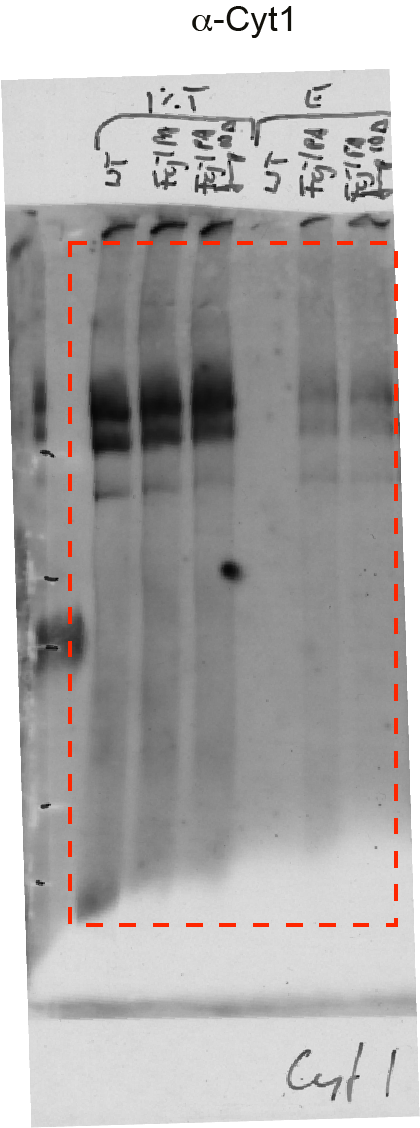

Supplement: Supplementary file 4 — Source data Fig. 2 [file 44319_2024_336_MOESM4_ESM.zip › Zerbes et al - source data Fig 2H.png]

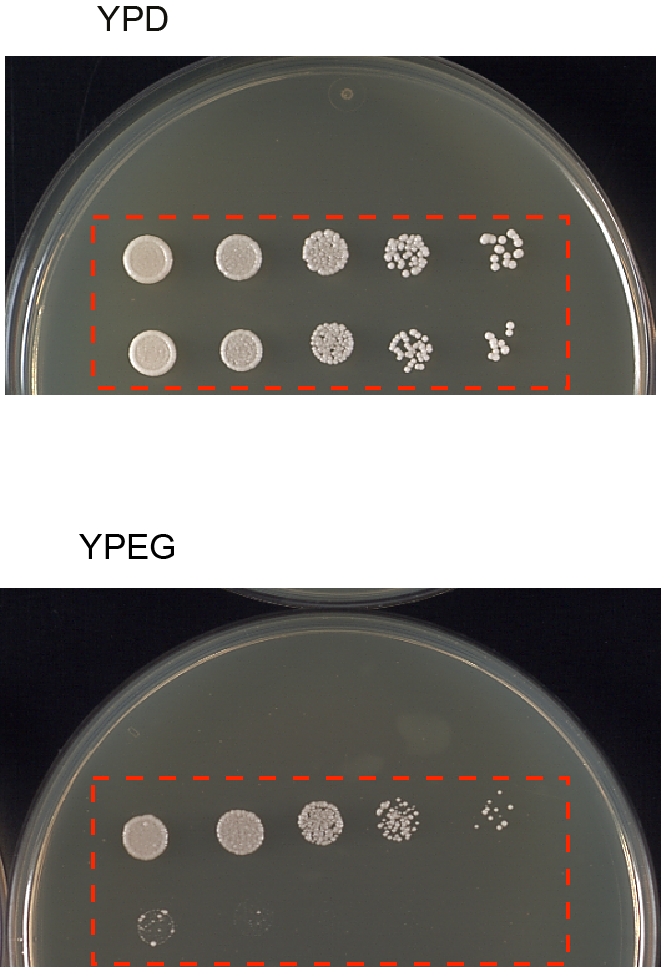

Supplement: Supplementary file 5 — Source data Fig. 3 [file 44319_2024_336_MOESM5_ESM.zip › Zerbes et al - source data Fig 3A.png]

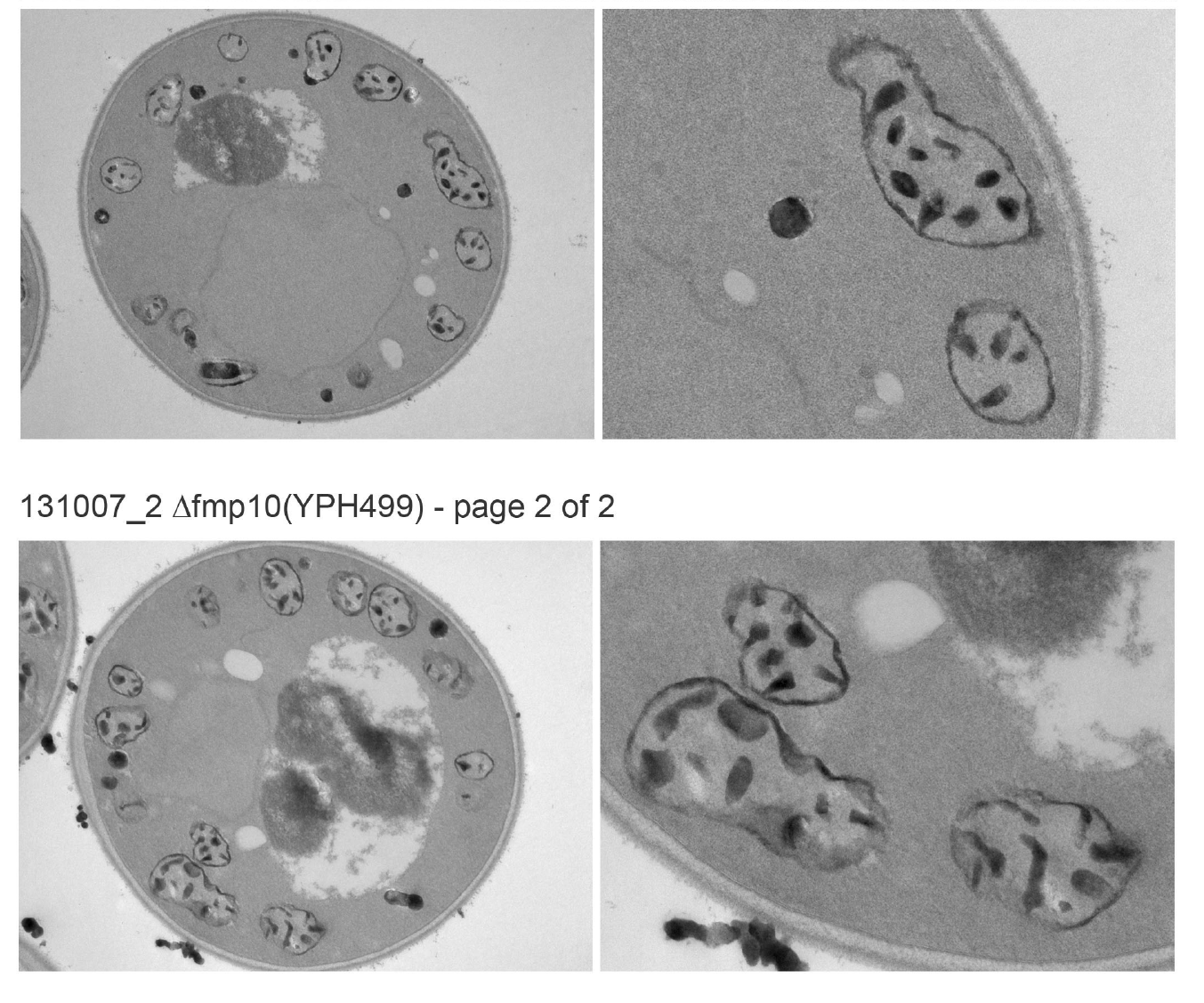

Supplement: Supplementary file 5 — Source data Fig. 3 [file 44319_2024_336_MOESM5_ESM.zip › Zerbes et al - source data Fig 3C.png]

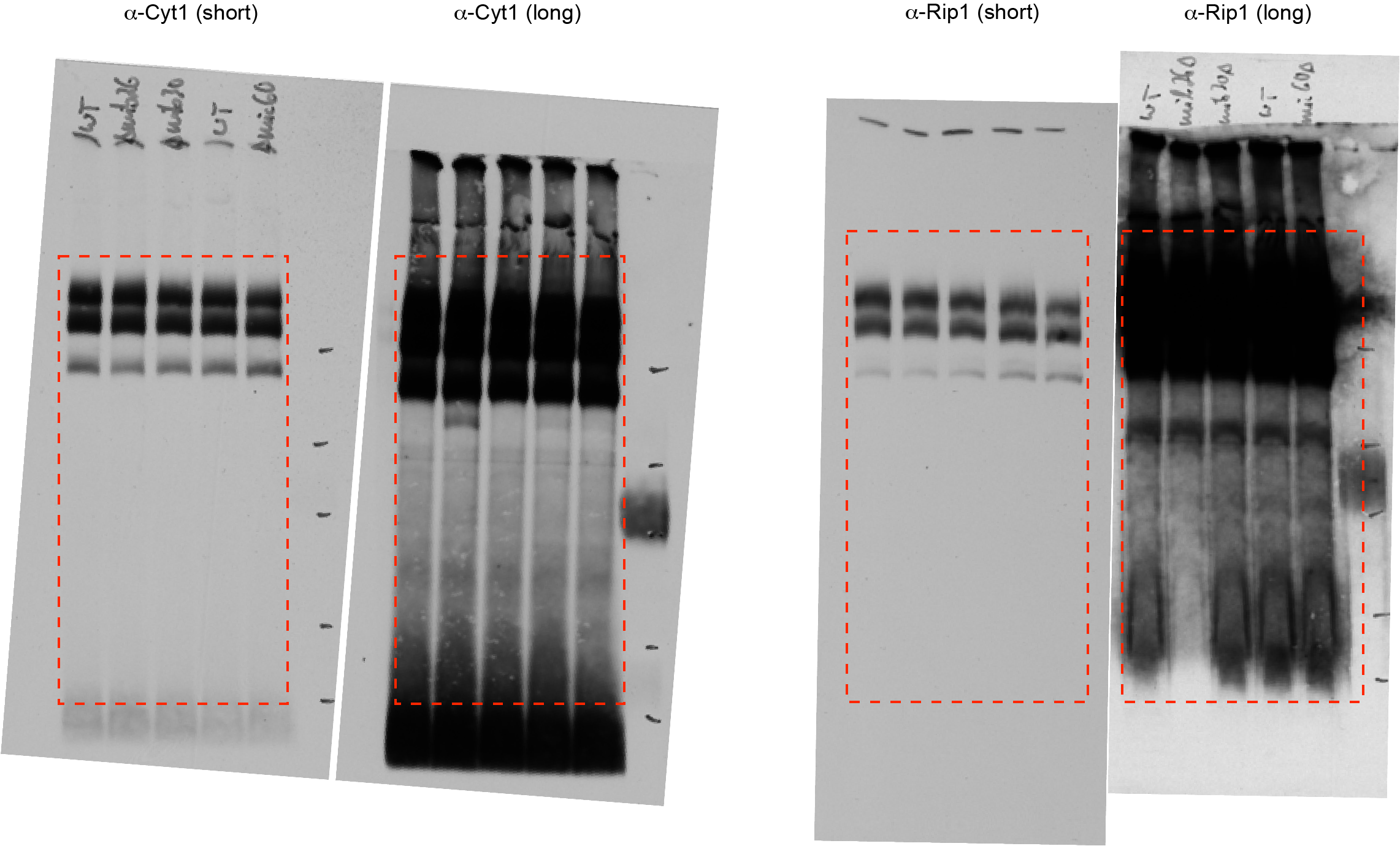

Supplement: Supplementary file 5 — Source data Fig. 3 [file 44319_2024_336_MOESM5_ESM.zip › Zerbes et al - source data Fig 3G.png]

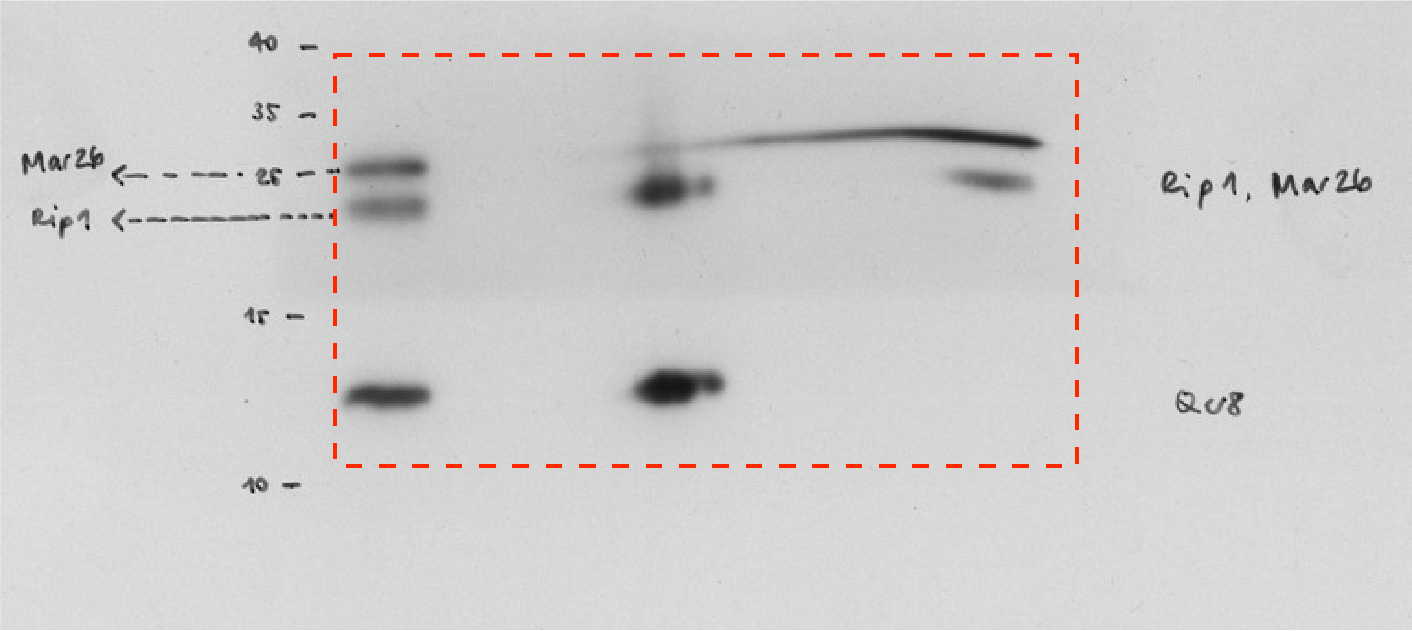

Supplement: Supplementary file 6 — Source data Fig. 4 [file 44319_2024_336_MOESM6_ESM.zip › Zerbes et al - source data Fig 4A.png]

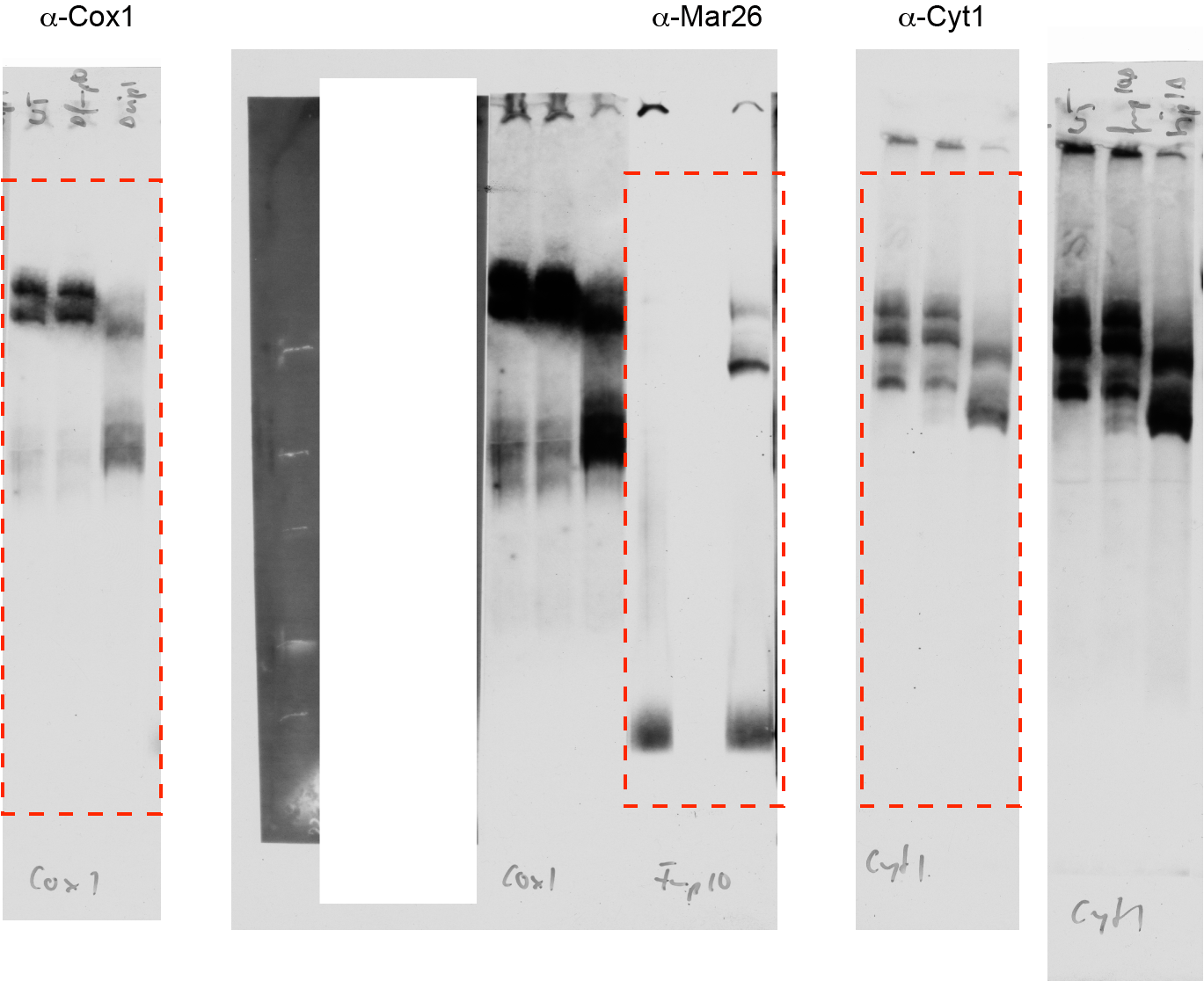

Supplement: Supplementary file 6 — Source data Fig. 4 [file 44319_2024_336_MOESM6_ESM.zip › Zerbes et al - source data Fig 4B.png]

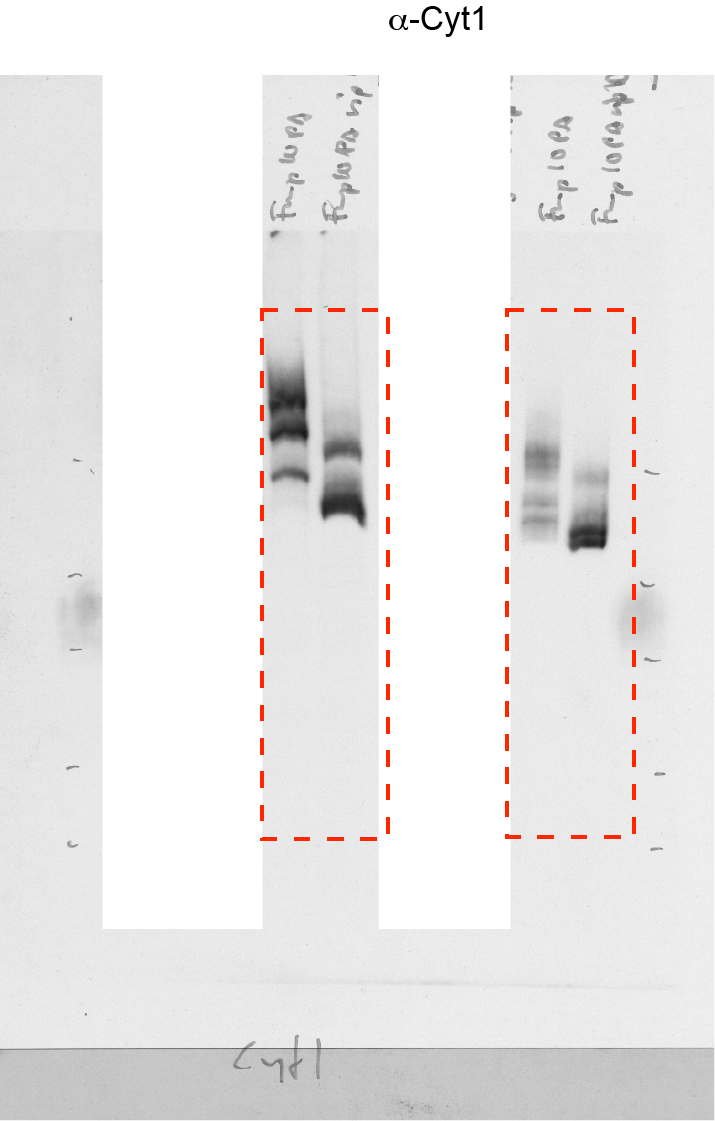

Supplement: Supplementary file 6 — Source data Fig. 4 [file 44319_2024_336_MOESM6_ESM.zip › Zerbes et al - source data Fig 4C.png]

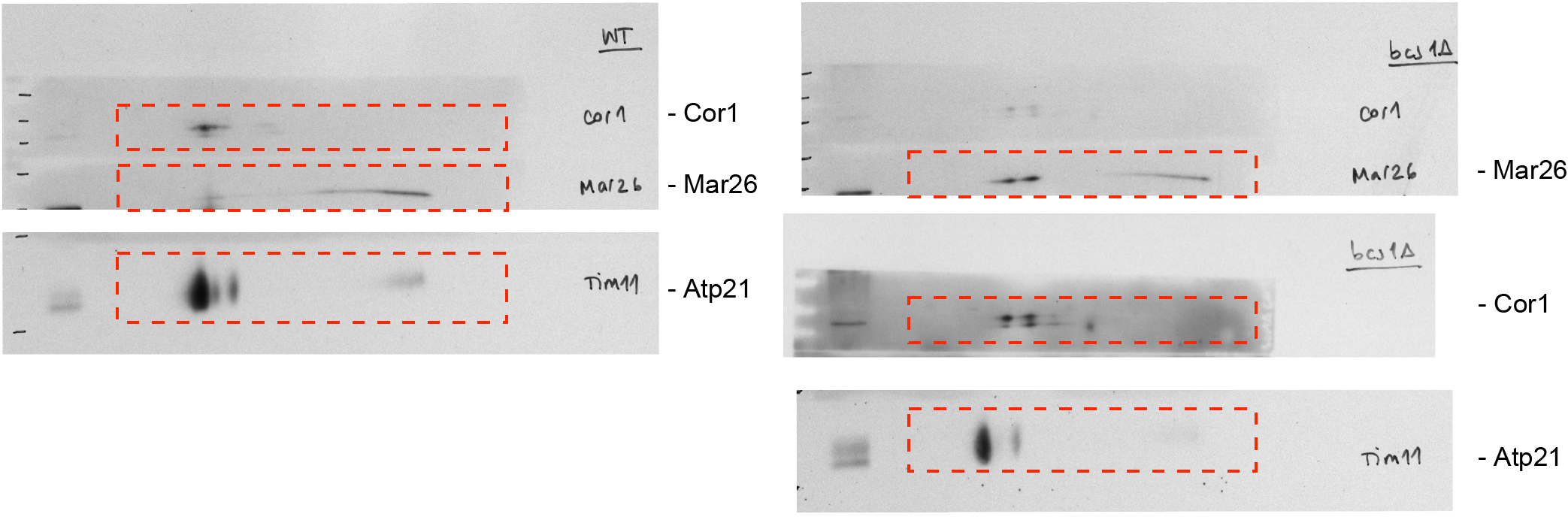

Supplement: Supplementary file 6 — Source data Fig. 4 [file 44319_2024_336_MOESM6_ESM.zip › Zerbes et al - source data Fig 4D.png]

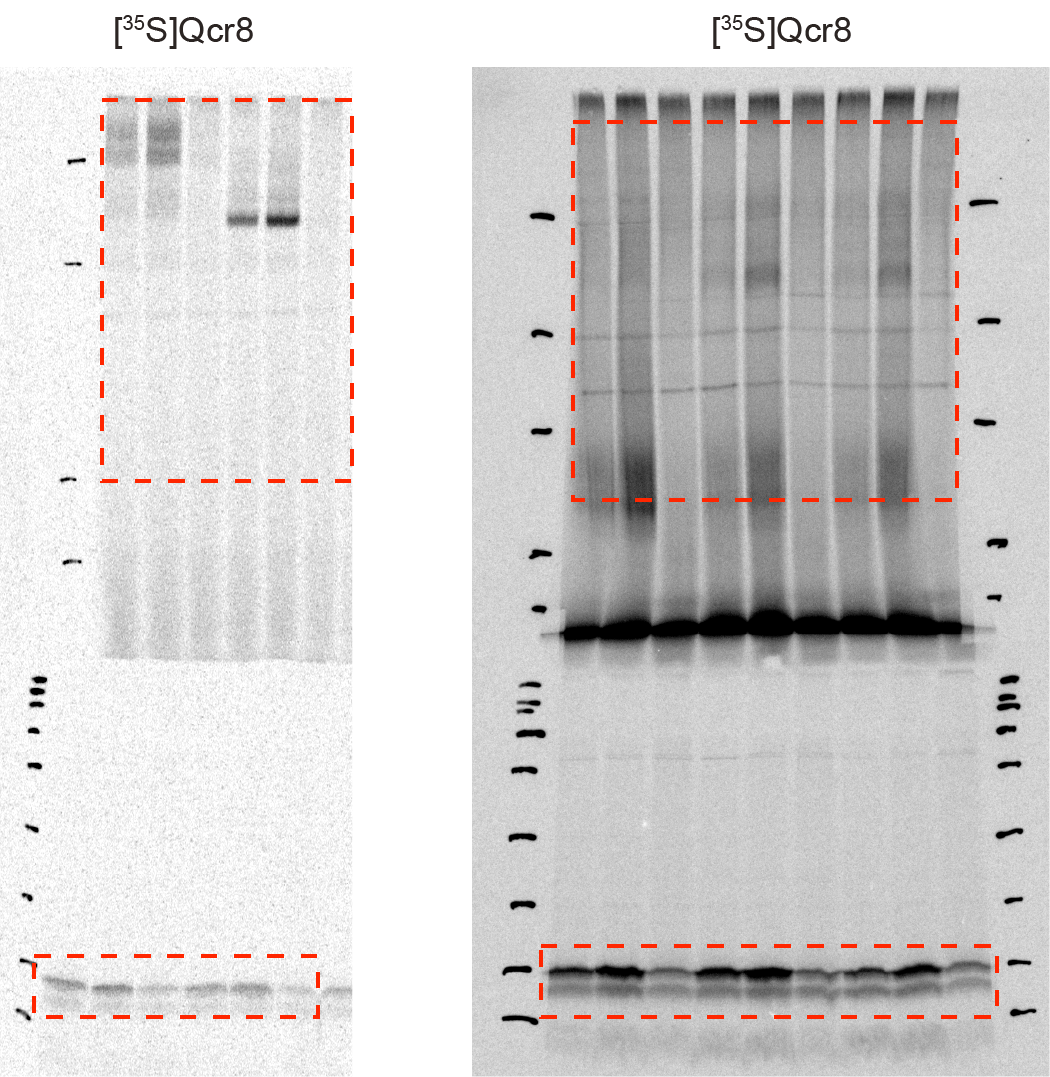

Supplement: Supplementary file 7 — Source data Fig. 5 [file 44319_2024_336_MOESM7_ESM.zip › Zerbes et al - source data Fig 5A.png]

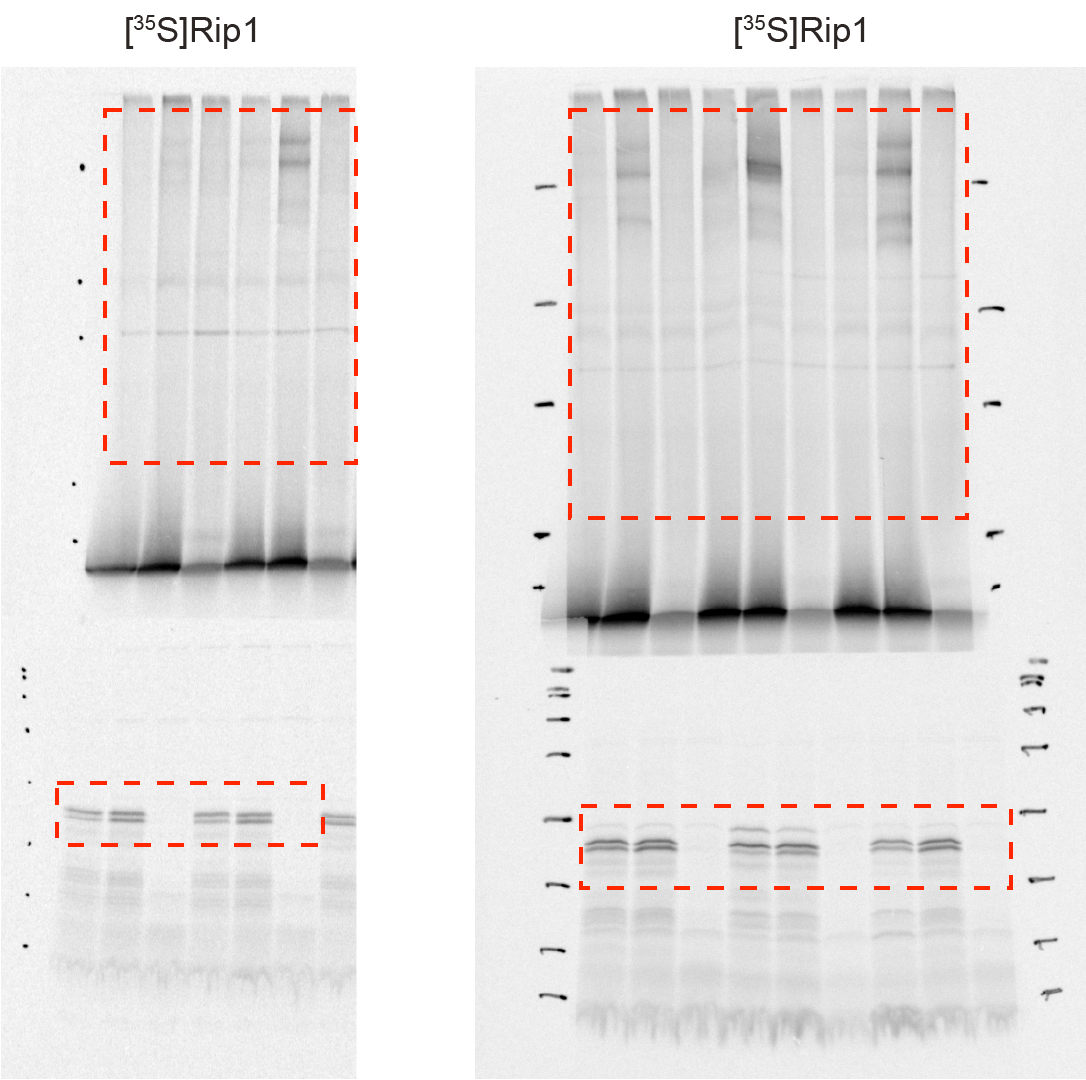

Supplement: Supplementary file 7 — Source data Fig. 5 [file 44319_2024_336_MOESM7_ESM.zip › Zerbes et al - source data Fig 5B.png]

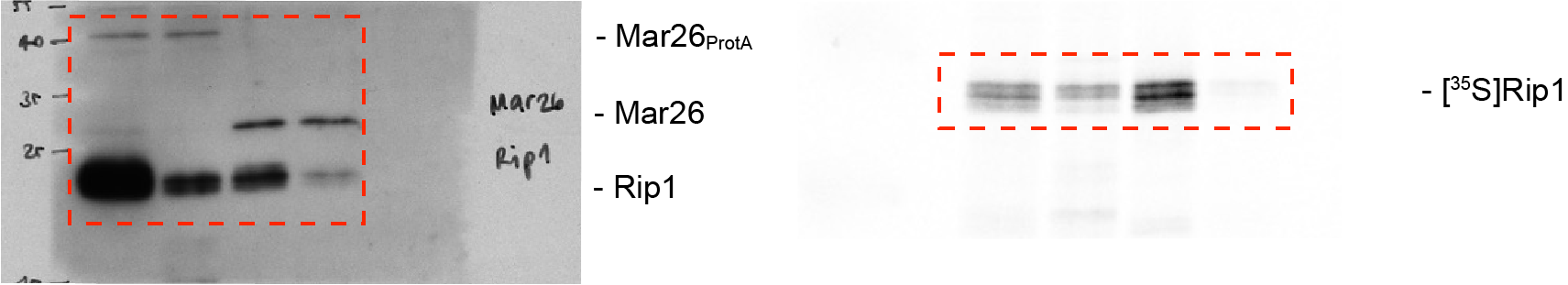

Supplement: Supplementary file 7 — Source data Fig. 5 [file 44319_2024_336_MOESM7_ESM.zip › Zerbes et al - source data Fig 5C.png]

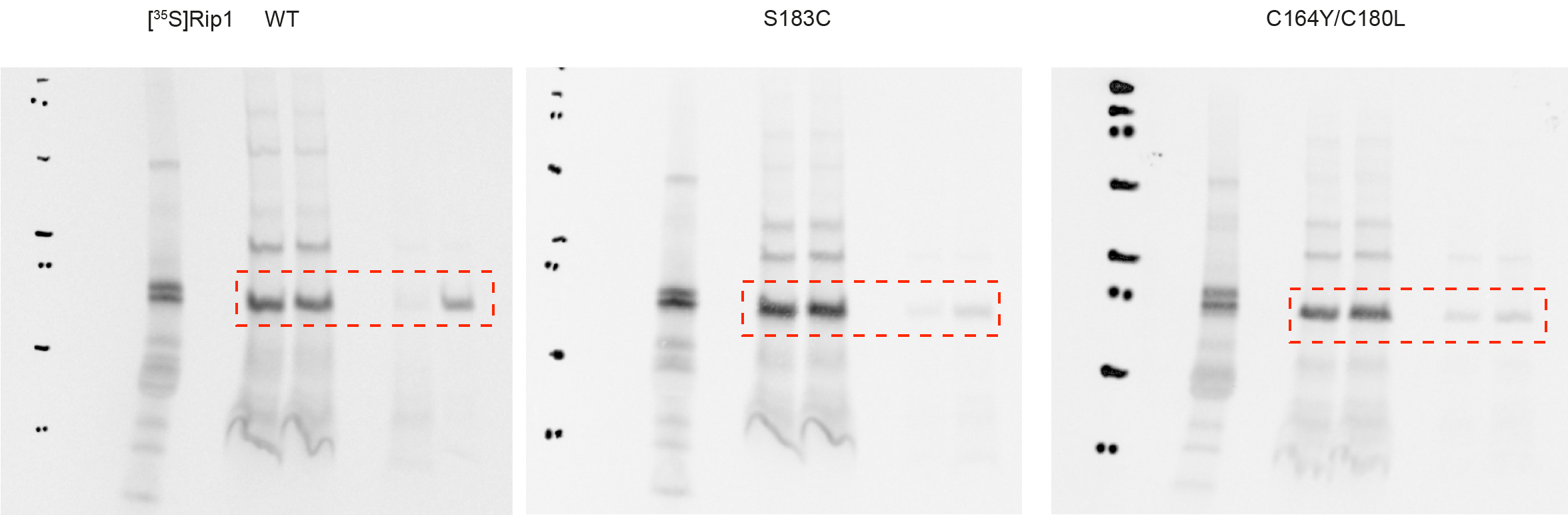

Supplement: Supplementary file 7 — Source data Fig. 5 [file 44319_2024_336_MOESM7_ESM.zip › Zerbes et al - source data Fig 5D.png]

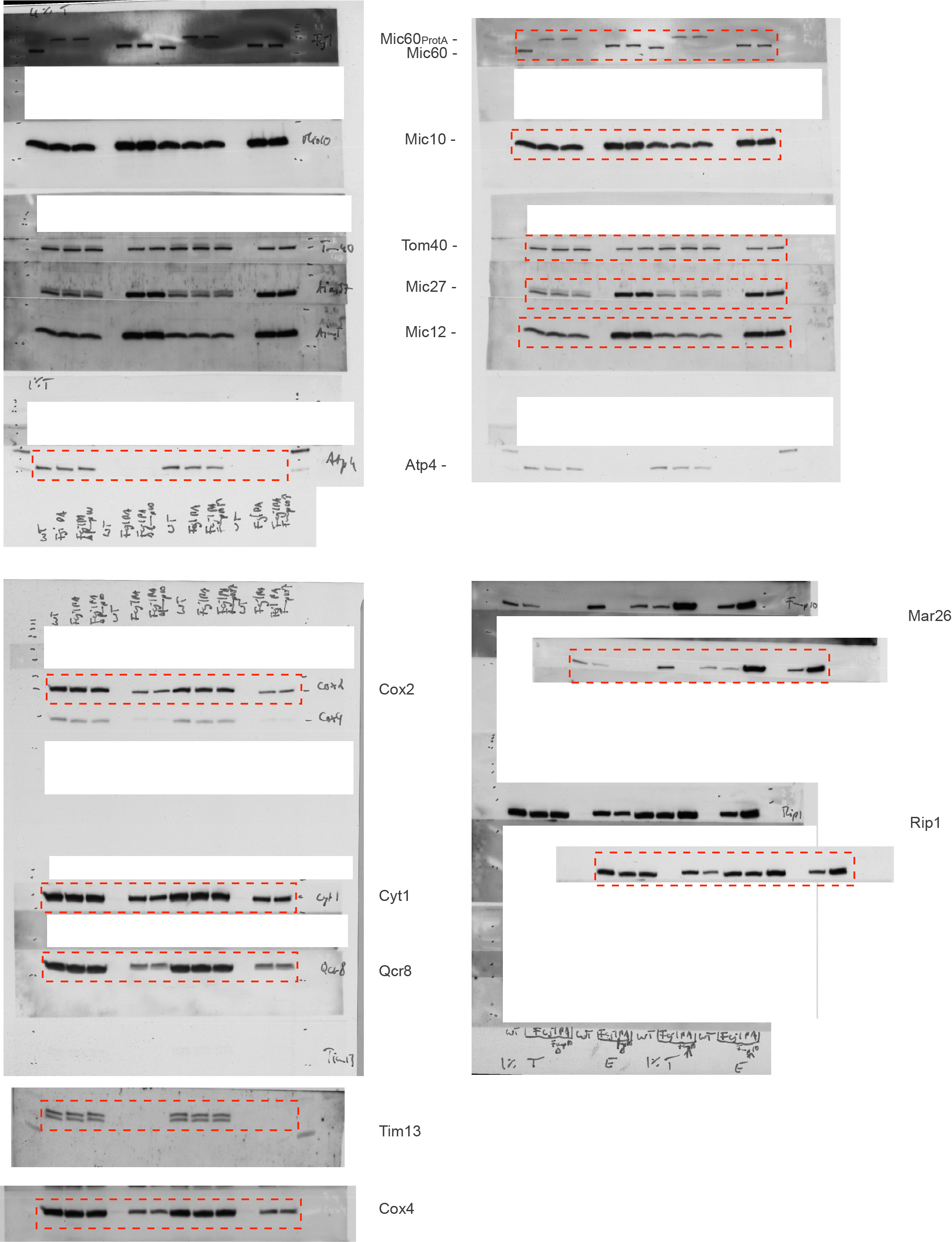

Supplement: Supplementary file 8 — Source data Fig. 6 [file 44319_2024_336_MOESM8_ESM.zip › Zerbes et al - source data Fig 6A.png]

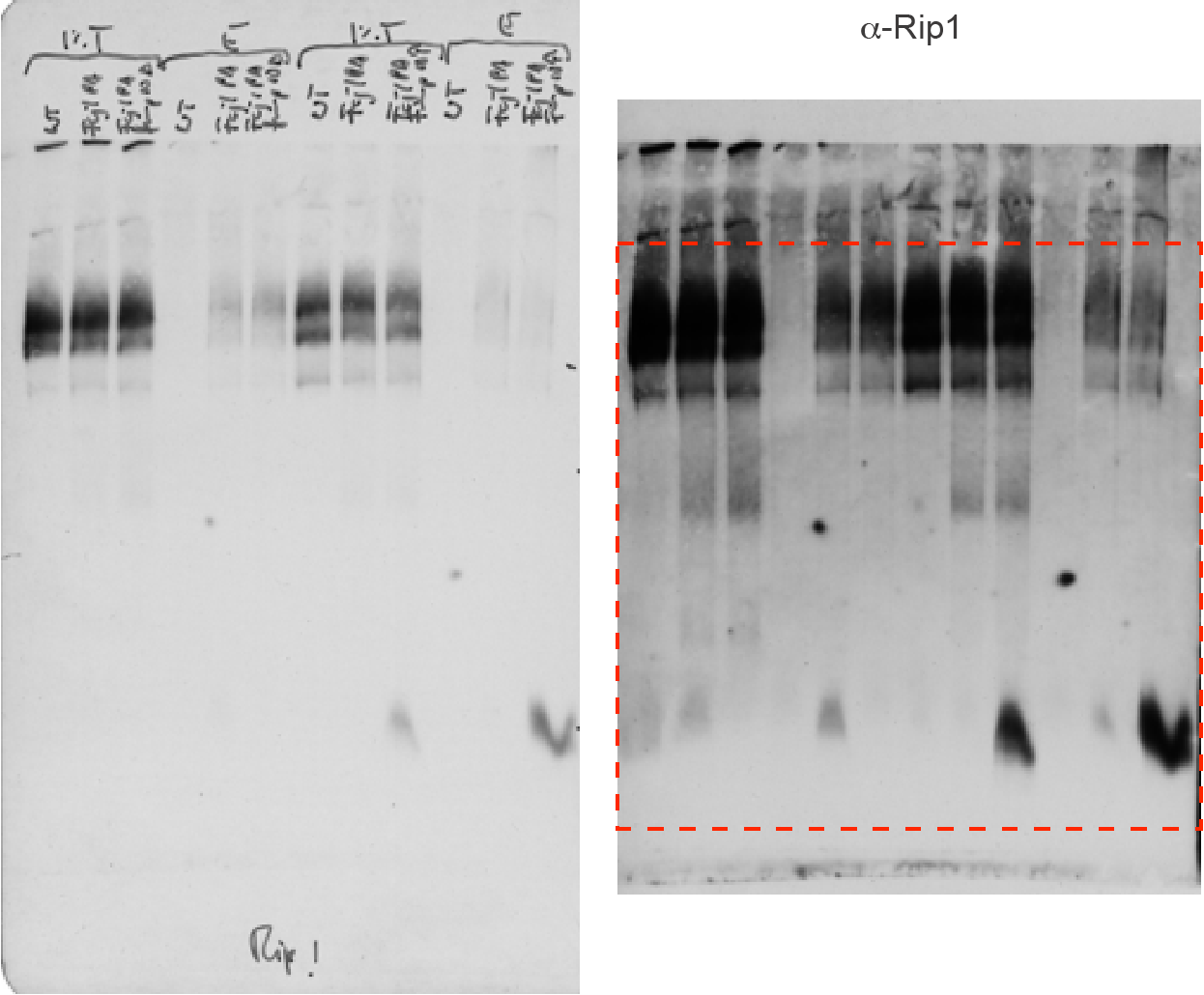

Supplement: Supplementary file 8 — Source data Fig. 6 [file 44319_2024_336_MOESM8_ESM.zip › Zerbes et al - source data Fig 6B.png]

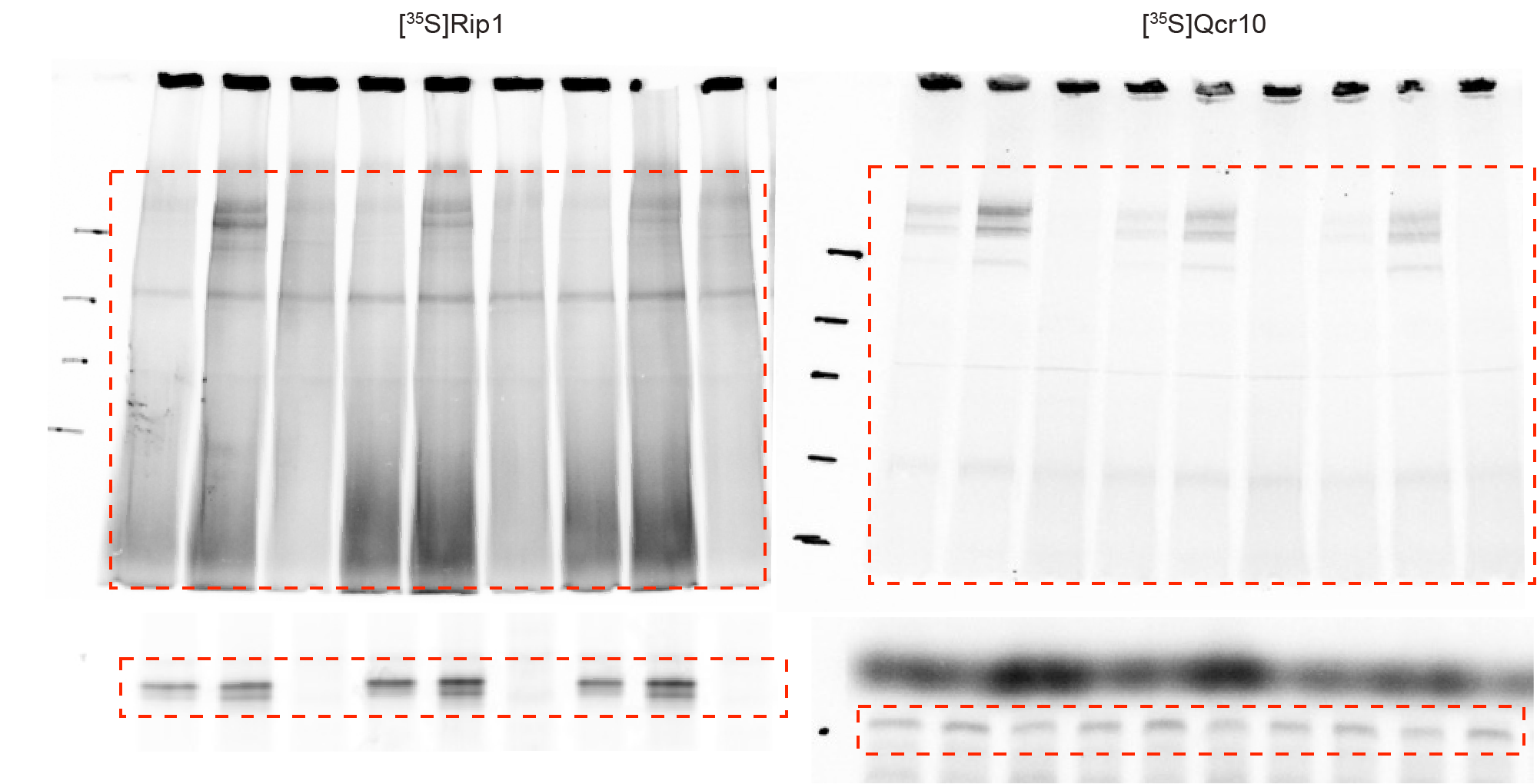

Supplement: Supplementary file 8 — Source data Fig. 6 [file 44319_2024_336_MOESM8_ESM.zip › Zerbes et al - source data Fig 6C.png]

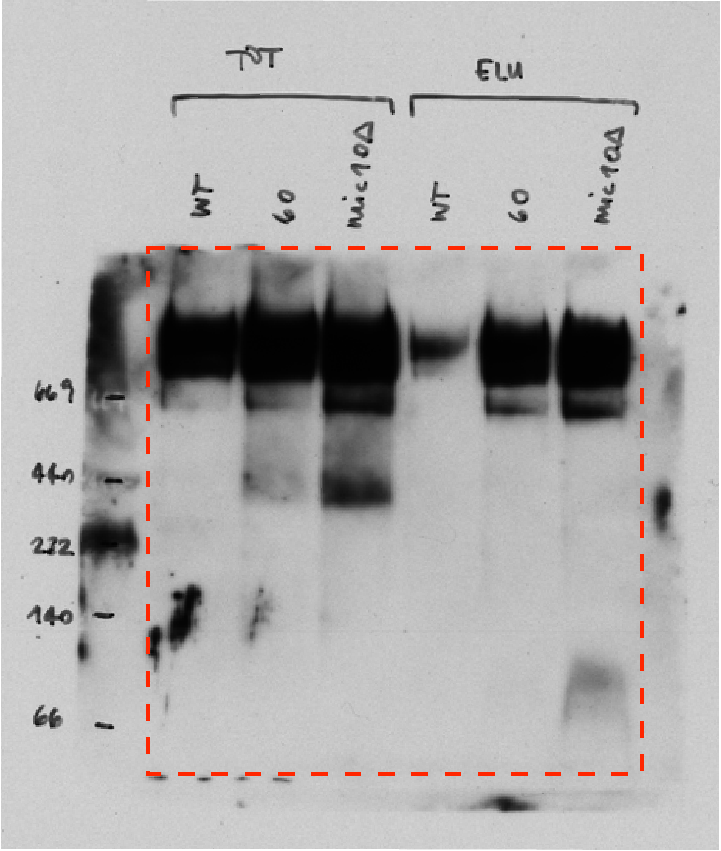

Supplement: Supplementary file 8 — Source data Fig. 6 [file 44319_2024_336_MOESM8_ESM.zip › Zerbes et al - source data Fig 6E.png]

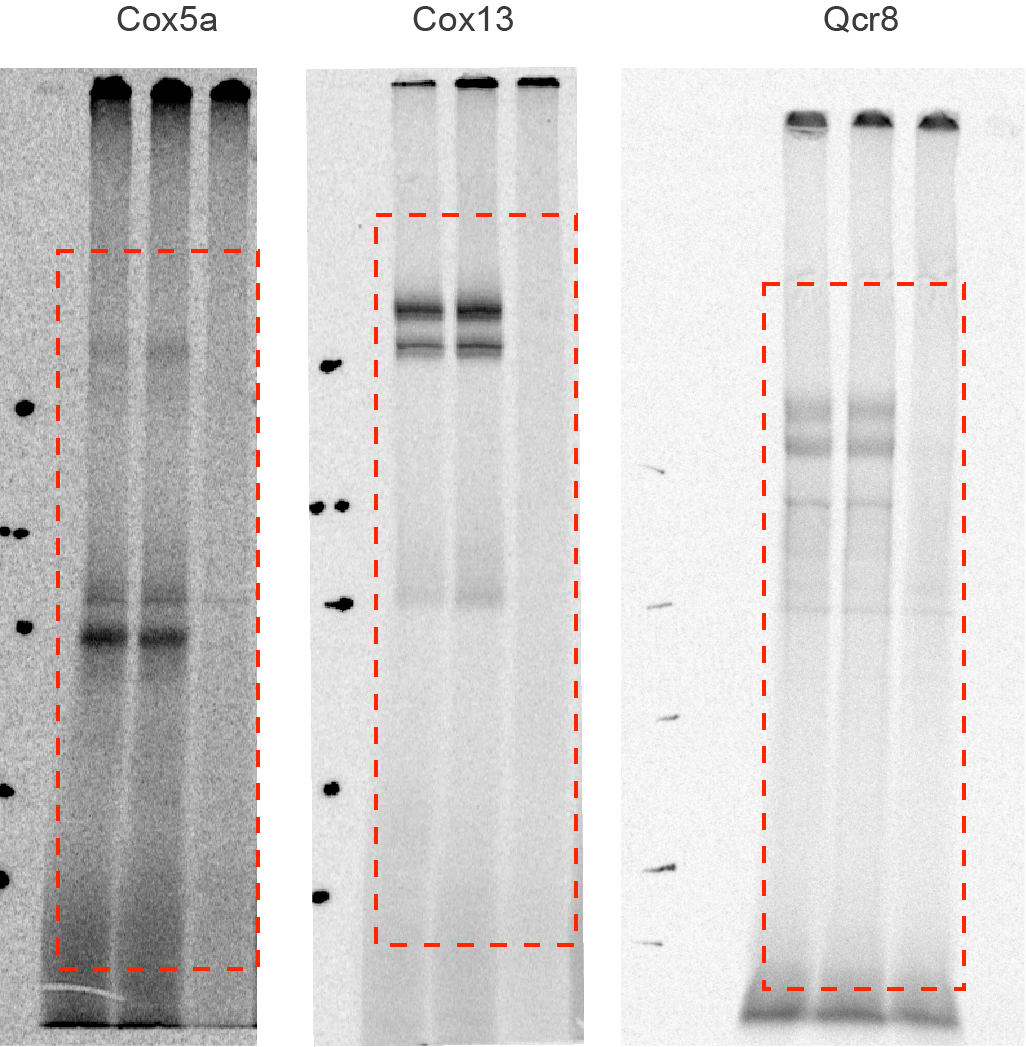

Supplement: Supplementary file 9 — Figure EV1 Source Data [file 44319_2024_336_MOESM9_ESM.zip › Zerbes et al - source data Fig EV1A.png]

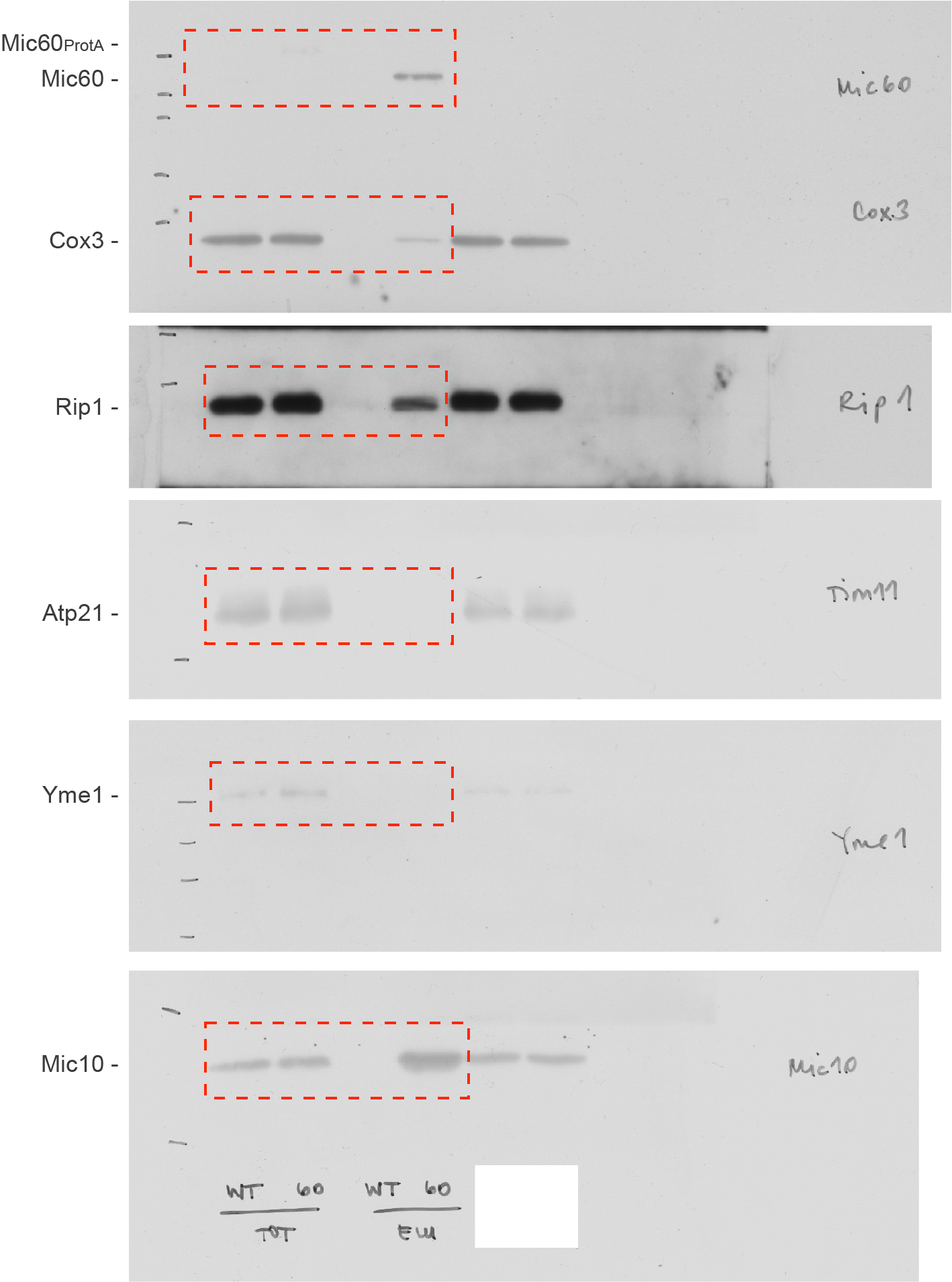

Supplement: Supplementary file 9 — Figure EV1 Source Data [file 44319_2024_336_MOESM9_ESM.zip › Zerbes et al - source data Fig EV1B.png]

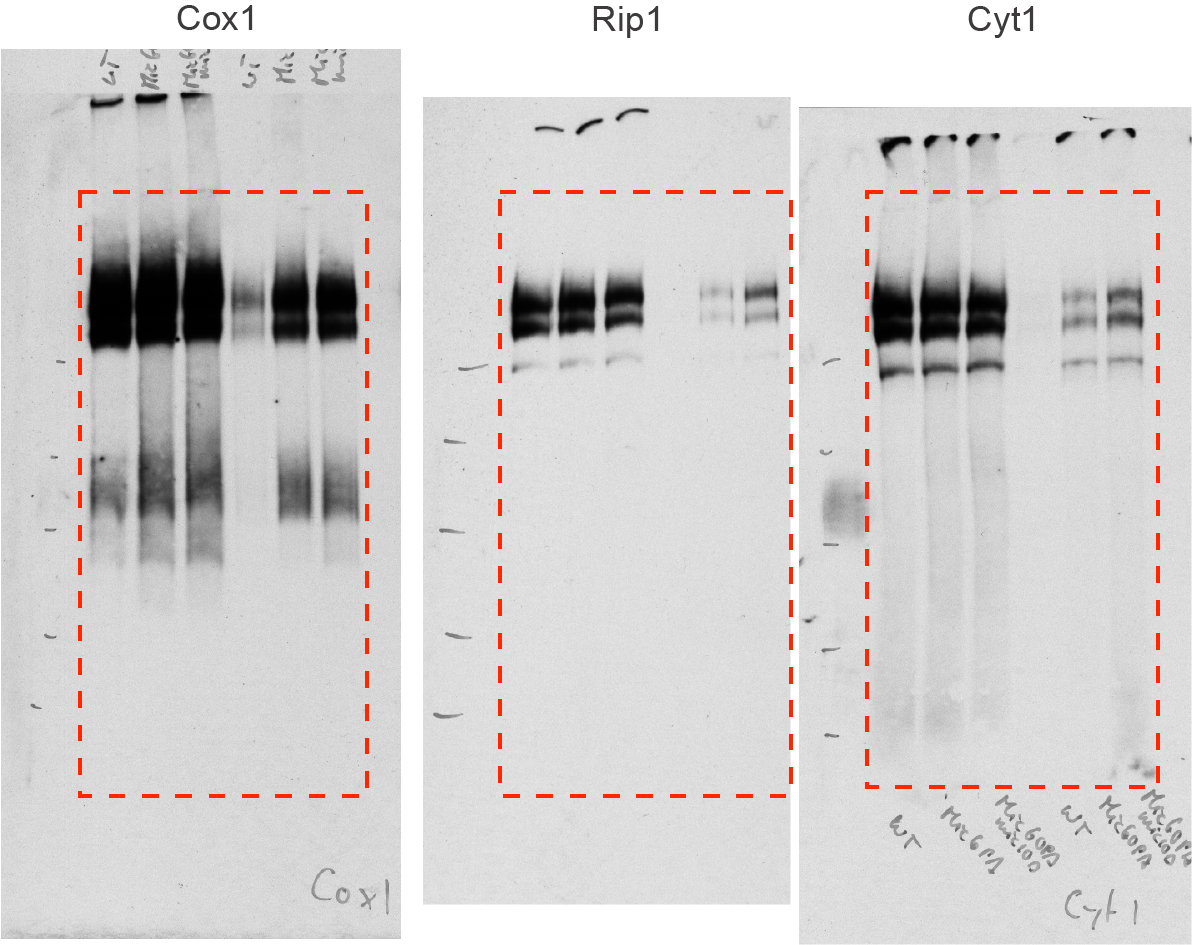

Supplement: Supplementary file 9 — Figure EV1 Source Data [file 44319_2024_336_MOESM9_ESM.zip › Zerbes et al - source data Fig EV1C.png]

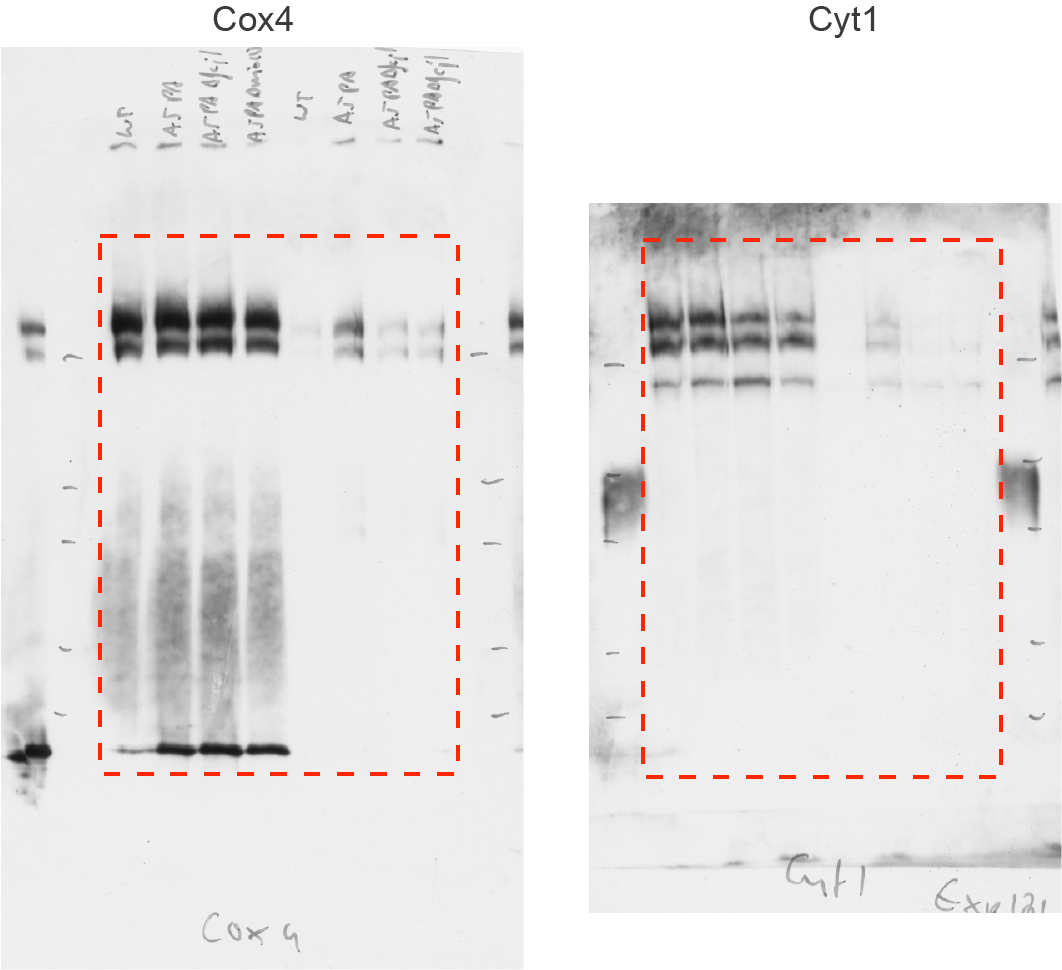

Supplement: Supplementary file 9 — Figure EV1 Source Data [file 44319_2024_336_MOESM9_ESM.zip › Zerbes et al - source data Fig EV1D.png]

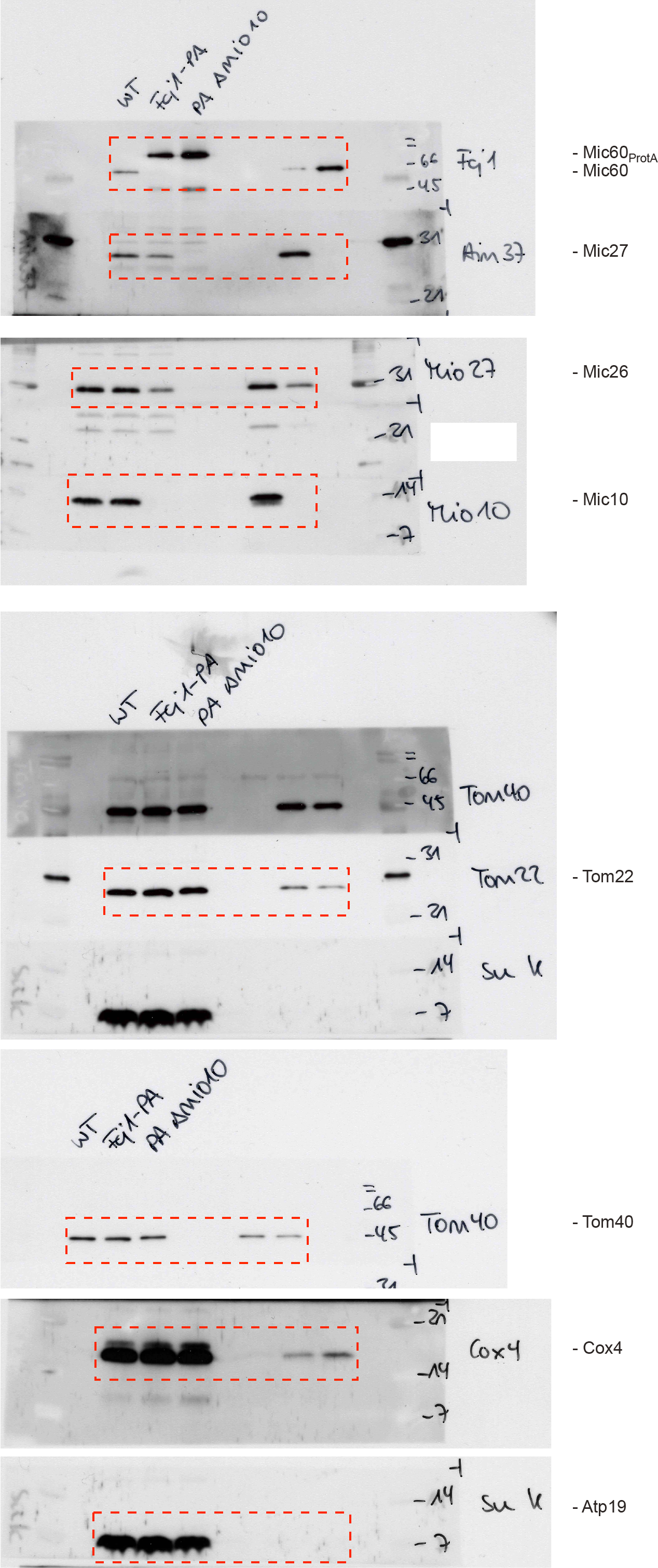

Supplement: Supplementary file 9 — Figure EV1 Source Data [file 44319_2024_336_MOESM9_ESM.zip › Zerbes et al - source data Fig EV1E.png]
