# Supplementary material for: Coordination of cytochrome bc1 complex assembly at MICOS
Source: EMBO Rep. 2024 Dec 2;26(2):353–84. doi: 10.1038/s44319-024-00336-x (PMC11772845; doi:10.1038/s44319-024-00336-x)
Supplement: Supplementary file 10 — Expanded View Figures [file 44319_2024_336_MOESM10_ESM.pdf]

Expanded View Figures

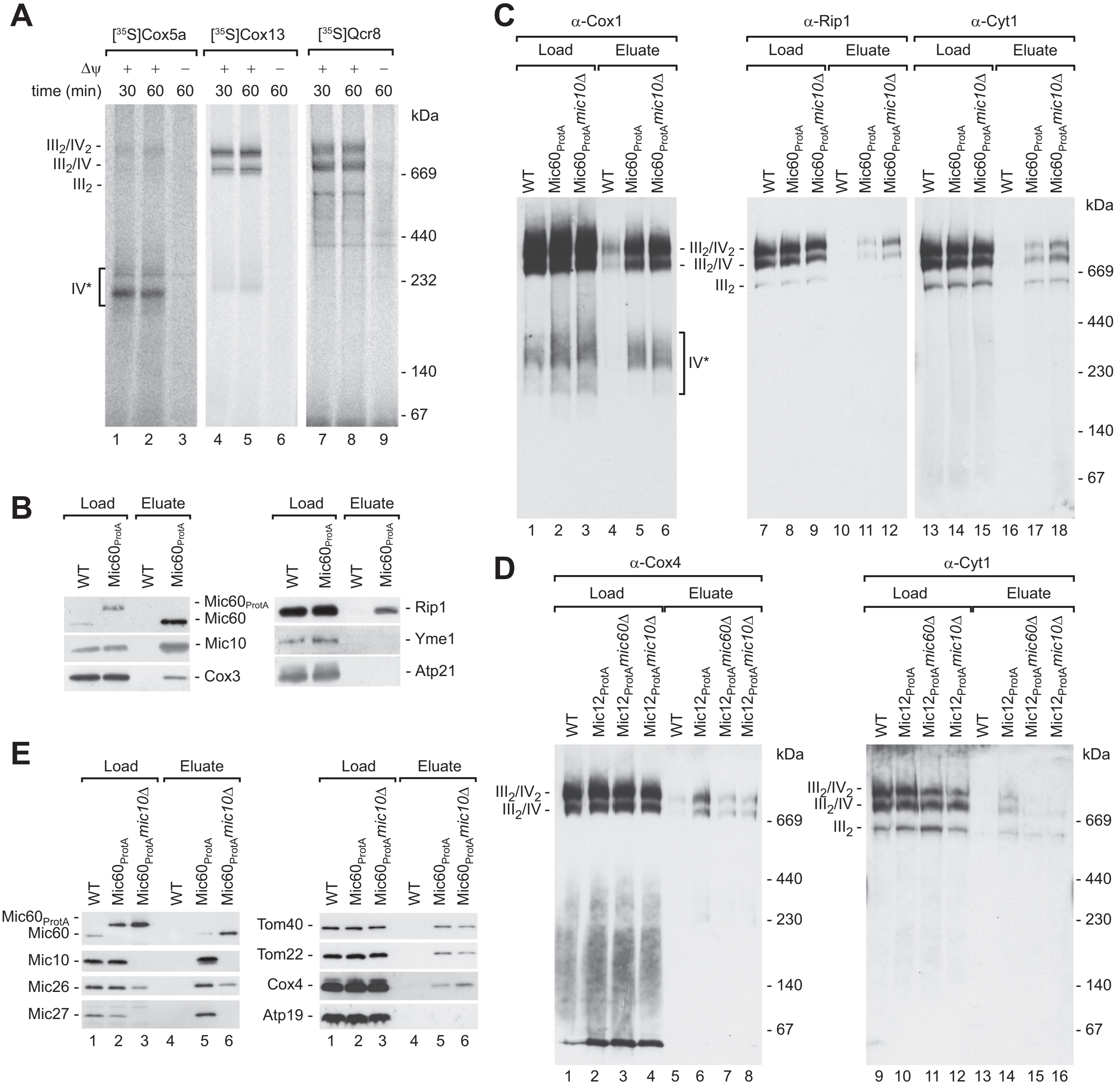

Figure EV1. The Mic60-Mic19 module mediates MICOS connection to the respiratory chain.

(**A**) The indicated radiolabeled preproteins were imported into wild-type mitochondria. Upon solubilization in digitonin-containing buffer, samples were analyzed by blue native (BN-) PAGE and autoradiography. III_2/IV_2, III_2/IV, III_2, supercomplexes of respiratory chain complexes III and IV; IV*, complex IV and assembly intermediates thereof; Δψ, membrane potential. (**B**) MICOS does not co-isolate the i-AAA protease Yme1 or the F_1F_o-ATP synthase subunit Atp21. IgG affinity purification was performed with wild-type (WT) and $Mic60_{ProtA}$ mitochondria and samples were analyzed by SDS-PAGE and immunoblotting. Load, 2%; Eluate, 100%. (**C**) Protein complexes were purified from digitonin-solubilized wild-type (WT), $Mic60_{ProtA}$ and $Mic60_{ProtA}$ *mic10Δ* mitochondria by IgG chromatography and analyzed by BN-PAGE and western blotting as in Fig. 1C. Load 1%, Eluate 100%. III_2/IV_2, III_2/IV, III_2, supercomplexes of respiratory chain complexes III and IV; IV*, complex IV and assembly intermediates thereof. (**D**) Protein complexes purified from wild-type, $Mic12_{ProtA}$, $Mic12_{ProtA}$ *mic60Δ* and $Mic12_{ProtA}$ *mic10Δ* mitochondria were analyzed as in (A). Load 1%, Eluate 100%. Cox4, complex IV subunit. (**E**) IgG purification was performed with wild-type (WT), $Mic60_{ProtA}$ and $Mic60_{ProtA}$ *mic10Δ* mitochondria. Samples were examined by SDS-PAGE and immunoblotting. Load, 4.3%; Eluate, 100%. Source data are available online for this figure.

© The Author(s)

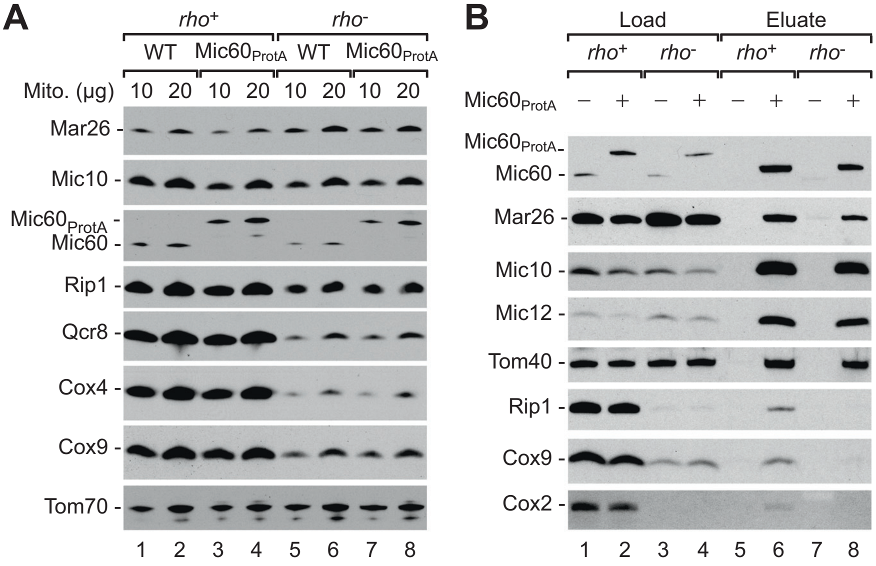

C

```
               10        20        30        40               50
Mar26  MFKRIAIAQIRTYTNGVVFKTASKPKRRWIPWTIFGGSFLGGW-------YLTQHMTFTD
       : . : .  ..  . .:.. ::.        .:.  :.:. .:        :.. .: ..
Mrx3   MSRTIPFL-FKLVNRAVILPTAG--------FTLGVGAFVKAWPDDAGVLSLNDPQTPAE
                10        20                30        40        50

            60        70         80        90       100       110
Mar26  LLAYWRYDALPKNADEV-VKYHADLNRRLNGLPIVKQLENAGFVQVIANEEENLLVSRAL
       :..  . .  : . ..: .  . . ..  : :   ...... : . : . ...  :...:
Mrx3   LISATK-SRQPMELQRVDILAQIEKSEVYNKLAQDEKMHHVLFSEKIPSGHREYHVGQGL
               60        70        80        90       100       110

             120       130       140       150       160        170
Mar26  NT-PGGVAIPPRVYYNPSRRETVGLYHLGMKLTGYPFLIHGGILATVIED-LMKEAIRLE
           : . : : :... .. : . .:::: .: .    .: :.:. .... :   .. :
Mrx3   LFGKGKLEIDPLVFHDVNHGELTVIYHLGAELGNRDGNVHKGLLSLLLDEALCYCGFPLL
              120       130       140       150       160       170

              180       190       200               210       220
Mar26  KGTKNINQETKNLSISYKFPTLANQFVVVRTTDLQQY--------GNKTKLKAELMDQSG
        . ...   :  ::. . :  .  . ...  .....         :.  ..  :. ...:
Mrx3   PSKRGV---TARLSLEF-FEDIPVDTTIILKANVKEIKGRKCIIEGHLEQFPLEVSSRNG
                 180        190       200       210       220

                          230       240
Mar26  NRT--------------LVKANATFSSEQGNPKEEK--------
       .:.              ..:  :  .    .:   :
Mrx3   TRSWNLPHIWGFNHKQEMAKKFAKANCILVEPTWFKYFKWLDMF
        230       240       250       260       270
```

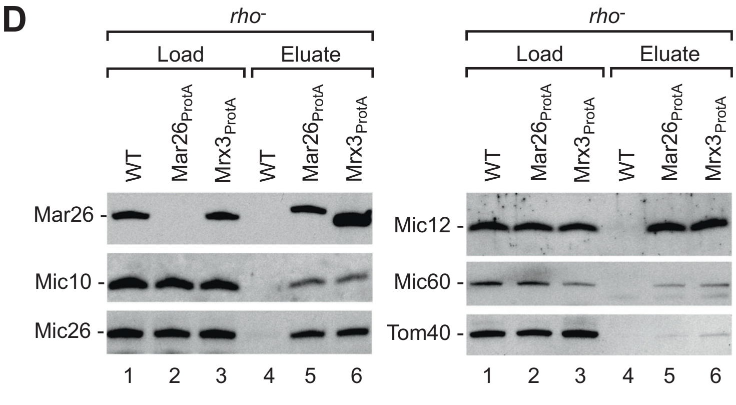

Figure EV2. Mar26 and Mrx3 are paralogs and interact with MICOS in the absence of a functional respiratory chain.

(**A**) Steady-state protein levels of *rho*$^{+}$ or *rho*$^{-}$ mitochondria isolated from wild-type (WT) cells or cells expressing $Mic60_{ProtA}$ were analyzed by SDS-PAGE and western blotting with the indicated antibodies. (**B**) Protein complexes were purified from *rho*$^{+}$ or *rho*$^{-}$ WT or $Mic60_{ProtA}$ mitochondria by IgG chromatography and analyzed by SDS-PAGE and western blotting. Load, 1%; Eluate 100%. (**C**) Alignment of the amino acid sequences of Mar26 and Mrx3. (**D**) Protein complexes were purified from WT, $Mar26_{ProtA}$ and $Mrx3_{ProtA}$ mitochondria isolated from *rho*$^{-}$ cells by IgG chromatography and analyzed by SDS-PAGE and western blotting. Load, 1%; Eluate 100%.

 © The Author(s)

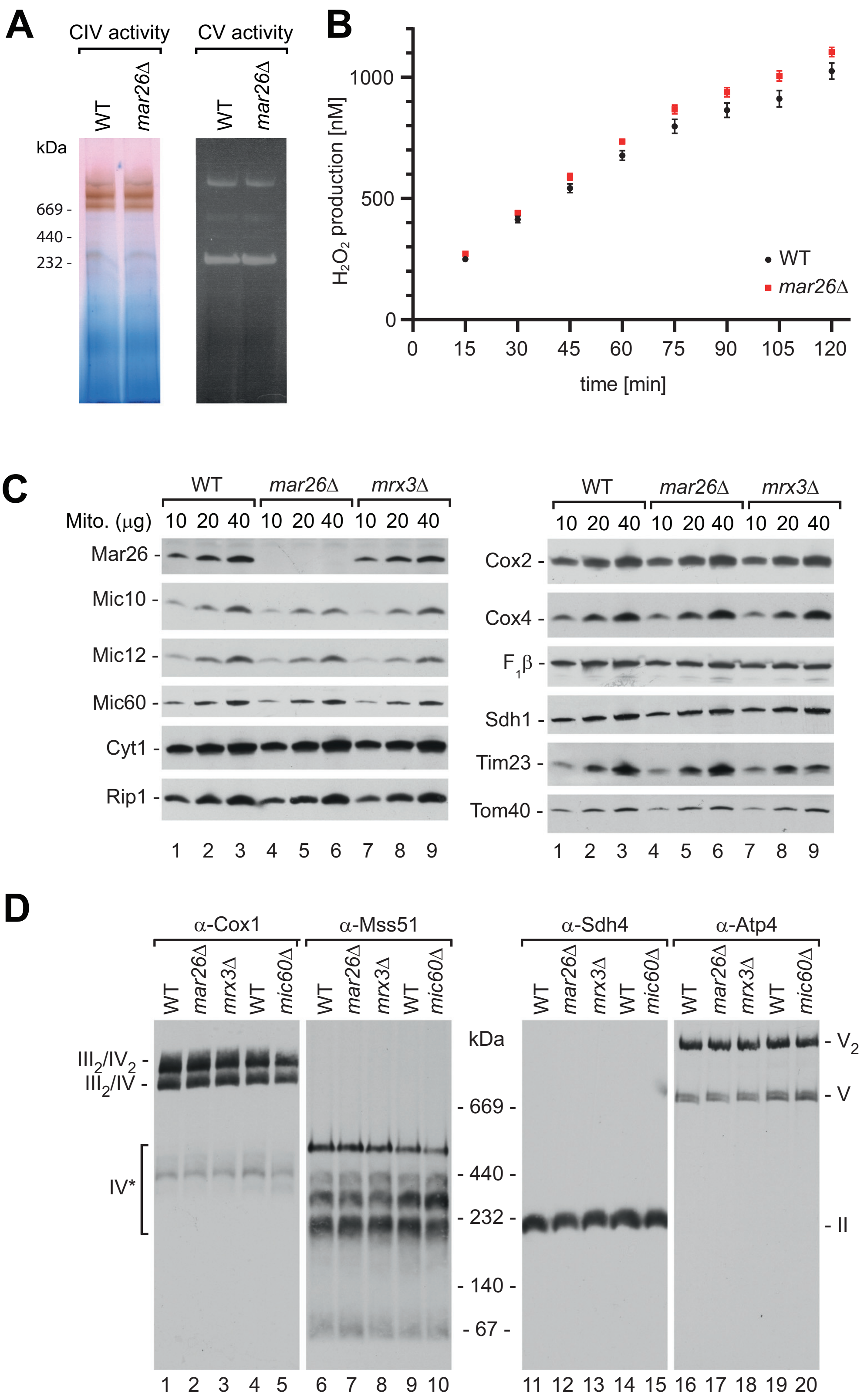
A
CIV activity
CV activity
WT
mar26Δ
kDa
669 -
440 -
232 -
B
H2O2 production [nM]
time [min]
WT
mar26Δ
C
WT
mar26Δ
mrx3Δ
Mito. (µg)
10 20 40 10 20 40 10 20 40
Mar26 -
Mic10 -
Mic12 -
Mic60 -
Cyt1 -
Rip1 -
Cox2 -
Cox4 -
F1β -
Sdh1 -
Tim23 -
Tom40 -
1 2 3 4 5 6 7 8 9
D
α-Cox1
α-Mss51
α-Sdh4
α-Atp4
WT
mar26Δ
mrx3Δ
WT
mic60Δ
III2/IV2 -
III2/IV -
IV*
kDa
- 669 -
- 440 -
- 232 -
- 140 -
- 67 -
- V2
- V
- II
1 2 3 4 5 6 7 8 9 10
11 12 13 14 15 16 17 18 19 20

Figure EV3. Mitochondrial protein or complex levels and activities in the absence of Mar26.

(**A**) Wild-type or *mar26Δ* mitochondria were solubilized and protein complexes were separated by BN-PAGE, followed by the measurement of in gel activities for complex IV (left panel) or ATPase activity of the F_1F_o-ATP synthase complexes (complex V, right panel). (**B**) In vivo H_2O_2 levels in WT and *mar26Δ* cells were detected over a time period of 120 min at 30 °C using Amplex Red. H_2O_2 levels were calculated using a H_2O_2 standard curve. Error bars: SEM; $n = 5$ (technical replicates). (**C**) Steady-state protein levels of WT, *mar26Δ* and *mrx3Δ* mitochondria were analyzed by SDS-PAGE and western blotting. Sdh1, complex II subunit. (**D**) WT, *mar26Δ*, *mrx3Δ*, and *mic60Δ* mitochondria were solubilized in digitonin buffer. Protein complexes were subsequently separated by BN-PAGE and detected by western blotting and immunodecoration with the indicated antibodies. III_2/IV_2, III_2/IV, supercomplexes of respiratory chain complexes III and IV; IV*, complex IV and assembly intermediates thereof; II, respiratory chain complex II (SDH); V_2, V, F_1F_o-ATP synthase (complex V) dimers and monomers, respectively.

 © The Author(s)

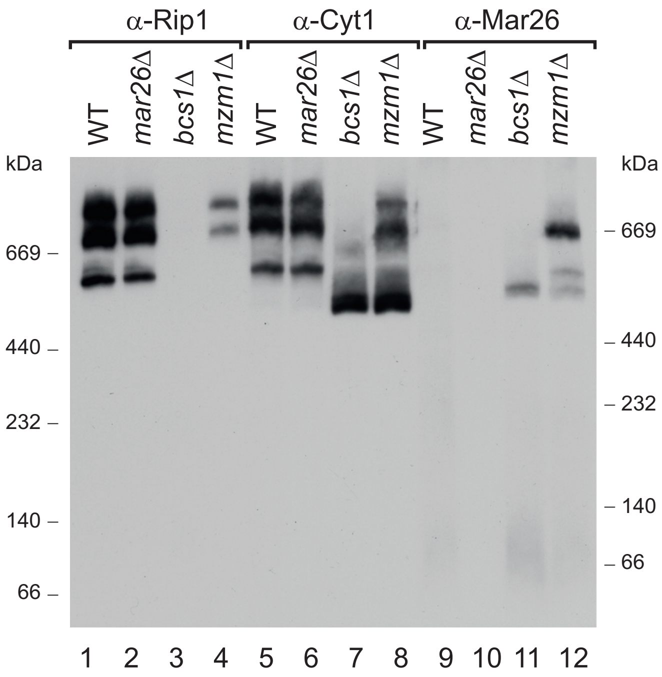

Figure EV4. Steady-state levels of mitochondrial protein complexes in *mar26Δ* mutant mitochondria.

Native protein complexes of wild-type, *mar26Δ*, *bcs1Δ* and *mzm1Δ* mitochondria were analyzed by western blotting against the indicated proteins.

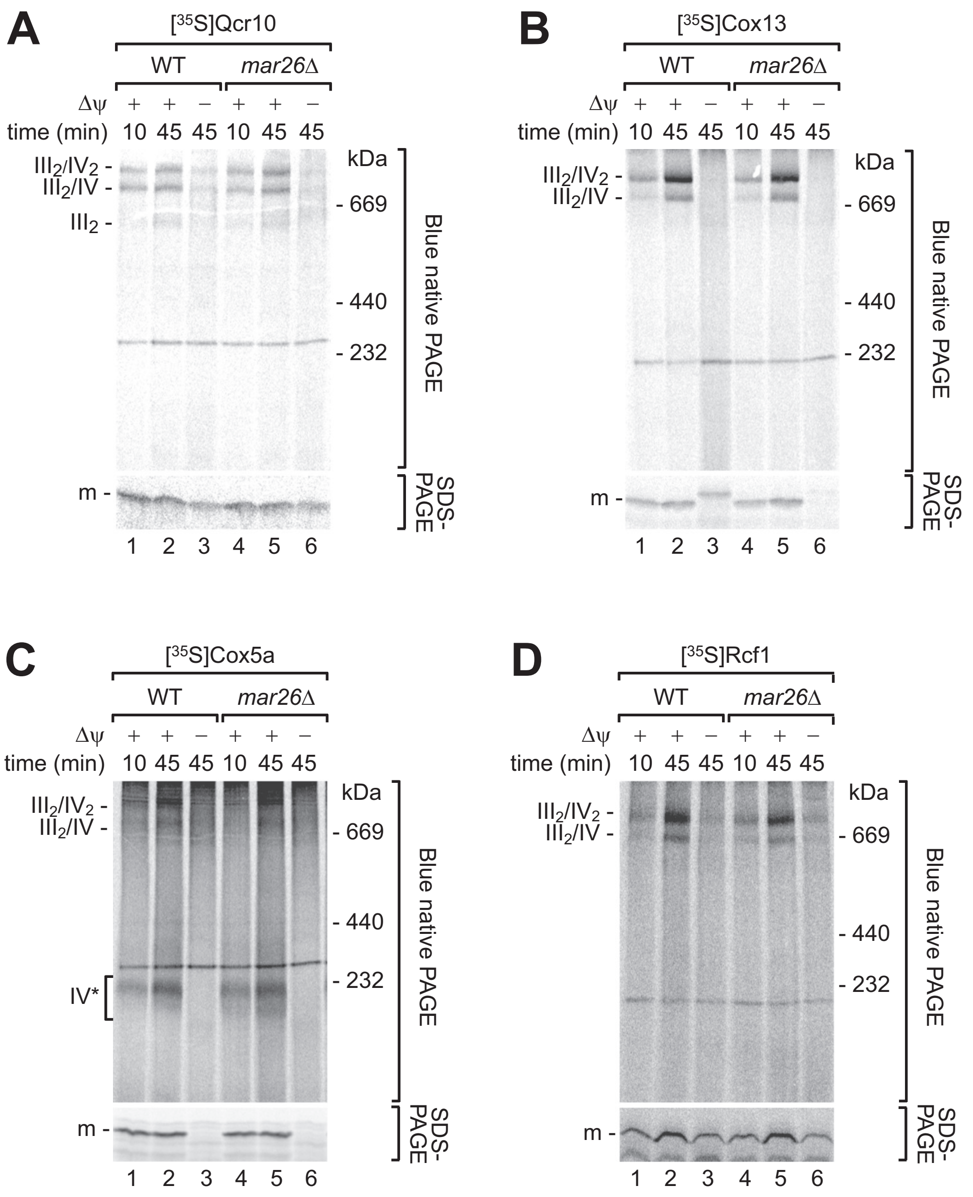

Figure EV5. Assembly of respiratory chain complex subunits in *mar26*Δ mitochondria.

Radiolabeled Qcr10 (**A**), Cox13 (**B**), Cox5a (**C**) or Rcf1 (**D**) preproteins were imported into wild-type or *mar26*Δ mitochondria in $Mic60_{ProtA}$ background. Samples were solubilized in digitonin buffer and analyzed by BN-PAGE or SDS-Page as indicated. Proteins were visualized by autoradiography. III_2/IV_2, III_2/IV, III_2, supercomplexes formed by respiratory chain complexes III and IV; IV*, assembly intermediates of complex IV; m, mature proteins; Δψ, membrane potential.

 © The Author(s)

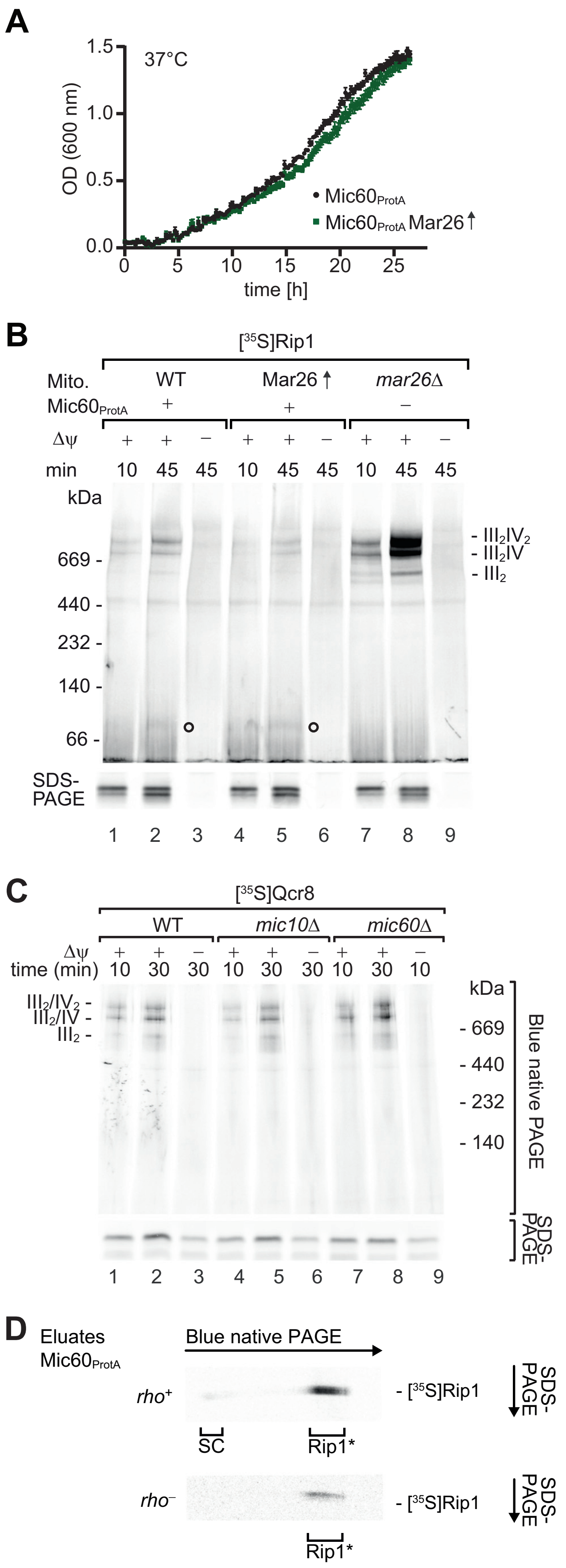
A
1.5
1.0
0.5
0.0
37°C
OD (600 nm)
0
5
10
15
20
25
time [h]
Mic60ProtA
Mic60ProtA Mar26↑
B
[35S]Rip1
Mito.
WT
Mar26↑
mar26Δ
Mic60ProtA
+
+
–
Δψ
min
10 45 45 10 45 45 10 45 45
kDa
669 -
440 -
232 -
140 -
66 -
- III2IV2
- III2IV
- III2
SDS-PAGE
1 2 3 4 5 6 7 8 9
C
[35S]Qcr8
WT
mic10Δ
mic60Δ
Δψ
time (min)
10 30 30 10 30 30 10 30 10
III2/IV2 -
III2/IV -
III2 -
kDa
- 669
- 440
- 232
- 140
Blue native PAGE
SDS-PAGE
1 2 3 4 5 6 7 8 9
D
Eluates Mic60ProtA
Blue native PAGE
rho+
- [35S]Rip1
SC
Rip1*
rho–
- [35S]Rip1
Rip1*
SDS-PAGE

Figure EV6. Consequences of Mar26 overexpression, and MICOS binding the Rip1 intermediate.

(**A**) $Mic60_{ProtA}$ and $Mic60_{ProtA}$ Mar26 overexpression (↑) cells were grown at 37 °C in liquid synthetic defined medium with 3% glycerol and 0.1% glucose as carbon sources. Error bars: SEM; $n = 8$ (2 independent experiments). (**B**) Radiolabeled Rip1 was imported into mitochondria isolated from $Mic60_{ProtA}$, $Mic60_{ProtA}$ Mar26 overexpression or *mar26*Δ cells. Mitochondria were subsequently solubilized in digitonin-containing buffer, analyzed by SDS-PAGE or BN-PAGE and visualized by autoradiography. Black circles, Rip1 intermediate; III_2/IV_2, III_2/IV, supercomplexes of respiratory chain complexes III and IV; Δψ, membrane potential. (**C**) Radiolabeled Qcr8 was imported into mitochondria isolated from wild-type (WT), *mic10*Δ or *mic60*Δ cells. The samples were analyzed as in (**B**) and visualized by autoradiography. (**D**) Radiolabeled Rip1 preprotein was imported for 60 min into mitochondria isolated from $Mic60_{ProtA}$ mitochondria of *rho*$^{+}$ or *rho*$^{-}$ background, Mic60 and interacting protein complexes were isolated by IgG chromatography, and the eluates were subjected to two-dimensional gel electrophoresis. To monitor the interaction of MICOS with complexes containing newly imported Rip1, the membranes were assessed by autoradiography.

 © The Author(s)
